# Serovar and sequence type distribution and phenotypic and genotypic antimicrobial resistance of *Salmonella* originating from pet animals in Chongqing, China

**DOI:** 10.1128/spectrum.03542-23

**Published:** 2024-05-17

**Authors:** Meiyuan Zhao, Xudong Wang, Jiawei He, Kexin Zhou, Mengqi Xie, Honglei Ding

**Affiliations:** 1 Laboratory of Veterinary Mycoplasmology, College of Veterinary Medicine, Southwest University, Chongqing, China; Institut National de Santé Publique du Québec, Sainte-Anne-de-Bellevue, Québec, Canada

**Keywords:** serovar, sequence type, AMR, MDR, ESBL, PMQR, QRDR

## Abstract

A total of 334 *Salmonella* isolates were recovered from 6,223 pet rectal samples collected at 50 pet clinics, 42 pet shops, 7 residential areas, and 4 plazas. Forty serovars were identified that included all strains except for one isolate that did not cluster via self-agglutination, with *Salmonella* Typhimurium monophasic variant, *Salmonella* Kentucky, *Salmonella* Enteritidis, *Salmonella* Pomona, and *Salmonella* Give being the predominant serovars. Fifty-one sequence types were identified among the isolates, and ST198, ST11, ST19, ST451, ST34, and ST155 were the most common. The top four dominant antimicrobials to which isolates were resistant were sulfisoxazole, ampicillin, doxycycline, and tetracycline, and 217 isolates exhibited multidrug resistance. The prevalence of β-lactamase genes in *Salmonella* isolates was 59.6%, and among these isolates, 185 harbored *bla*_TEM_, followed by *bla*_CTX-M_ (66) and *bla*_OXA_ (10). Moreover, six PMQR genes, namely, including *qnrA* (4.8%), *qnrB* (4.2%), *qnrD* (0.9%), *qnrS* (18.9%), *aac(6')-Ib-cr* (16.5%), and *oqxB* (1.5%), were detected. QRDR mutations (76.6%) were very common in *Salmonella* isolates, with the most frequent mutation in *parC* (T57S) (47.3%). Furthermore, we detected six tetracycline resistance genes in 176 isolates, namely, *tet*(A) (39.5%), *tet*(B) (8.1%), *tet*(M) (7.7%), *tet*(D) (5.4%), *tet*(J) (3.3%), and *tet*(C) (1.8%), and three sulfonamide resistance genes in 303 isolates, namely, *sul1* (84.4%), *sul2* (31.1%), and *sul3* (4.2%). Finally, we found 86 isolates simultaneously harboring four types of resistance genes that cotransferred 2–7 resistance genes to recipient bacteria. The frequent occurrence of antimicrobial resistance, particularly in dogs and cats, suggests that antibiotic misuse may be driving multidrug-resistant *Salmonella* among pets.

**IMPORTANCE:**

Pet-associated human salmonellosis has been reported for many years, and antimicrobial resistance in pet-associated *Salmonella* has become a serious public health problem and has attracted increasing attention. There are no reports of *Salmonella* from pets and their antimicrobial resistance in Chongqing, China. In this study, we investigated the prevalence, serovar diversity, sequence types, and antimicrobial resistance of *Salmonella* strains isolated from pet fecal samples in Chongqing. In addition, β-lactamase, QRDR, PMQR, tetracycline and sulfonamide resistance genes, and mutations in QRDRs in *Salmonella* isolates were examined. Our findings demonstrated the diversity of serovars and sequence types of *Salmonella* isolates. The isolates were widely resistant to antimicrobials, notably with a high proportion of multidrug-resistant strains, which highlights the potential direct or indirect transmission of multidrug-resistant *Salmonella* from pets to humans. Furthermore, resistance genes were widely prevalent in the isolates, and most of the resistance genes were spread horizontally between strains.

## INTRODUCTION

*Salmonella* is a well-known foodborne pathogen that colonizes humans and different animal species and is the second most commonly reported zoonotic etiological agent, causing an estimated 550 million diarrheal diseases worldwide annually, with 155,000 deaths (1). Moreover, the global burden of invasive non-typhoidal *Salmonella* was estimated to be 3.4 million cases and 681,316 deaths annually, with 63.7% of cases occurring in children younger than 5 years (2). To date, more than 2,600 *Salmonella* serovars have been identified based on the reactivity of antisera to somatic (O) and flagellar (H) antigens (3, 4), and numerous *Salmonella* serovars are known to infect both humans and animals (5). Usually, *Salmonella* is transmitted to humans through livestock (6), poultry (7), contaminated food products of animal origin (8–11), or the environment (8, 9).

Recently, there have been many reports of *Salmonella* carried by pets, including dogs (12–15), cats (13), lizards (16, 17), turtles (18), parrots (19), hedgehogs (20), snakes (21, 22), and domestic pigeons (23). Moreover, increasing evidence that *Salmonella* is transmitted to people directly through close contact with pet animals or indirectly through contact with the pet environment, contaminated surfaces, goods, and water, has been obtained in many parts of the world (18, 20, 24–26). These transmitted *Salmonella* strains cause diseases, especially in children under 5 years old, immunocompromised people, and elderly individuals (18, 20, 24–26).

Antimicrobial resistance (AMR) of *Salmonella* has become a serious public health problem in recent years, especially for strains with multidrug resistance (MDR), which is defined as acquired non-susceptibility to at least one agent in three or more antimicrobial categories (27). There have been many reports of infections caused by drug-resistant *Salmonella* in food animals (6, 7, 11, 28). Additionally, the AMR of pet-associated *Salmonella* has attracted increasing attention. Several studies have shown that many pet-associated *Salmonella* are resistant to antimicrobials, even newer generations of antibiotics, and these drug-resistant pet-associated *Salmonella* strains are transmitted to humans, causing serious public health problems (18, 20, 24–26). Furthermore, multiple resistance genes, including extended-spectrum β-lactamase (ESBL) genes and plasmid-mediated quinolone resistance (PMQR) genes, which were analyzed simultaneously with the quinolone resistance-determining region (QRDR) within the *gyrA* and *parC* genes, were sometimes detected in drug-resistant *Salmonella* isolates obtained from pets (29–31). ESBLs are a group of enzymes mediating resistance to most β-lactams approved for use in human and veterinary medicine, including extended-spectrum cephalosporins and monobactams but excluding carbapenems and cephamycins. The mechanism of quinolone resistance has been elucidated to involve PMQR and chromosomal mutations in QRDRs (32, 33). Three mechanisms of PMQR, namely, *qnr*, *aac(6')-Ib-cr*, and active efflux pumps, *oqxAB* and *qepA*, have been discovered in clinical isolates (33), whereas mutations in QRDR genes, which encode DNA gyrase or topoisomerase IV, are also frequently found in quinolone-resistant *Salmonella* isolates (32). Doxycycline, a tetracycline antibiotic, is widely used to treat bacterial diseases in pets. Several different *tet* (tetracycline resistance) genes have been described as conferring resistance to tetracyclines in bacteria, and studies have shown that *Salmonella* resistance to tetracycline or doxycycline is frequently due to the presence of *tet* genes in these bacteria (34–36). Sulfonamides are also widely used to treat bacterial infections in humans and domestic animals but not in pet animals in China (37, 38). Resistance to sulfonamides occurs principally through the acquisition of the alternative dihydropteroate synthase (DHPS) gene *sul*, the product of which has a low affinity for sulfonamides (38). Unlike resistance to other classes of antimicrobials, such as tetracycline, which is encoded by many different genes, only four known sulfonamide resistance (*sul*) genes (*sul1*, *sul2*, *sul3,* and *sul4*) have been identified (39–42).

Close physical contact or coexistence in the same domestic environment between pet animals and humans promotes the pet-to-human transmission of bacteria (18, 20, 24, 25). Improving living standards and increased urbanization in Chongqing have led to an increase in the frequency of urban pets in this area (43). However, there are reports of antimicrobial-resistant *Salmonella* from pets elsewhere in China and abroad (12–17, 22, 23, 31, 44, 45). Therefore, in this study, we investigated the prevalence, serovar and sequence type (ST) distribution, and antimicrobial susceptibility of *Salmonella* strains isolated from pets in Chongqing. In addition, we dissected the relationship between phenotypes and genotypes regarding resistance to β-lactams, quinolones, tetracyclines, and sulfonamides and explored the transmission of identified resistance genes in highly resistant isolates.

## RESULTS

### *Salmonella* isolation frequency

A total of 334 *Salmonella* isolates were obtained from 6,223 rectal swabs (5.4%) collected from pet animals at 50 pet clinics, 42 pet shops, 7 residential areas, and 4 plazas (Table S1). Of these 334 isolates, 159 (4.4%, 159/3638) were recovered from dogs, 90 (3.7%, 90/2409) were recovered from cats, 71 (81.6%, 71/87) were recovered from turtles, 14 (66.7%, 14/21) were recovered from lizards, and no isolate was acquired from any samples collected from parrots, hedgehogs, pet rabbits, pet rats, foxes, or a snake (Table S2). Overall, the isolation rate among turtles was greater than that from other sources (χ^2^*P* < 0.001). Moreover, a significantly greater prevalence of *Salmonella* was detected in lizards than in dogs and cats (*P* < 0.001). There was no difference in the isolation rate between dogs and cats (*P* ≥ 0.05). Statistically, the isolation rates of *Salmonella* in dogs (6.9%, 26/376) and cats (7.2%, 29/401) with diarrhea were much greater than those in dogs (4.0%, 132/3262) and cats (3.1%, 62/2008) without diarrhea (dog: *P* < 0.05; cats: *P* < 0.001). No diarrhea was observed in the other sampled animals.

### *Salmonella* serovar distribution

Except for one isolate that did not exhibit self-agglutination in sterile saline, the other 333 strains were classified into 40 serovars (Table 1; Table S3). Among the serotyped organisms, *Salmonella* Typhimurium monophasic variant (11.7%, 39/334), *Salmonella* Kentucky (10.2%, 34/334), *Salmonella* Enteritidis (8.7%, 29/334), *Salmonella* Pomona (6.9%, 23/334), and *Salmonella* Give (6.0%, 20/334) were most frequently isolated and accounted for 43.4% (145/334) of the total number of isolates. To our knowledge, this was the first report of serovars Takoradi, Bokanjac, II Lethe, 61:z:z44, 23:z:-, 17:a:z35, 18:z23:-, 23:-:6, 45:a:z35, 50:-:-, 52:-:1,5,7, and 60:r:- in China, and most of these serovars were isolated from reptiles. For example, *Salmonella* 23:z:- and *Salmonella* 17:a:z35 were isolated from turtles; *Salmonella* 45:a:z35, *Salmonella* 50:-:-, and *Salmonella* 52:-:1,5,7 were recovered from lizards; and Bokanjac was obtained from both turtles (83.3%, 5/6) and a lizard (16.7%, 1/6). *Salmonella* II Lethe, *Salmonella* 61:z:z44, *Salmonella* 18:z23:-, and *Salmonella* 23:-:6 were isolated from dogs and turtles. *Salmonella* Takoradi was isolated from dogs and turtles, and serovar 60:r:- was solely isolated from a dog without diarrhea. The distribution of serovars varied among sources, Typhimurium monophasic variant, Kentucky, and Enteritidis were the predominant serovars originating from dogs and cats, accounting for 40.9% (65/159) and 41.1% (37/90) of the respective strains. The dominant serovars of strains from turtles and lizards were Pomona (25.4%, 18/71) and Cotham (64.3%, 9/14), respectively. We did not find a relationship between the distribution of serovars and season.

**TABLE 1:** Serovars of *Salmonella* isolated from pets in Chongqing, China

Serovar	Dogs (%)	Cats (%)	Turtles (%)	Lizards (%)	Total (%)
Typhimurium monophasic variant	25 (15.7)	14 (15.6)			39 (11.7)
Kentucky	22 (13.8)	12 (13.3)			34 (10.2)
Enteritidis	18 (11.3)	11 (12.2)			29 (8.7)
Pomona	1 (0.6)	4 (4.4)	18 (25.4)		23 (6.9)
Give	13 (8.2)	7 (7.8)			20 (6.0)
Saintpaul	6 (3.8)	5 (5.6)	4 (5.6)		15 (4.5)
Mbandaka	13 (8.2)	1 (1.1)			14 (4.2)
Typhimurium	10 (6.3)	4 (4.4)			14 (4.2)
Cotham		1 (1.1)	3 (4.2)	9 (64.3)	13 (3.9)
Bovismorbificans	8 (5.0)	2 (2.2)	1 (1.4)		11 (3.3)
Rissen	8 (5.0)	3 (3.3)			11 (3.3)
Takoradi	4 (2.5)	7 (7.8)			11 (3.3)
Derby	6 (3.8)	4 (4.4)			10 (3.0)
61:z:z44	4 (2.5)		5 (7.0)		9 (2.7)
Stanley	5 (3.1)	1 (1.1)	2 (2.8)		8 (2.4)
Kottbus			6 (8.5)	1 (7.1)	7 (2.1)
23:z:-			6 (8.5)		6 (1.8)
Abony	2 (1.3)	3 (3.3)	1 (1.4)		6 (1.8)
Bokanjac			5 (7.0)	1 (7.1)	6 (1.8)
Chester	2 (1.3)		3 (4.2)		5 (1.5)
Essen	1 (0.6)	4 (4.4)			5 (1.5)
Manhattan			5 (7.0)		5 (1.5)
Cerro	1 (0.6)	3 (3.3)			4 (1.2)
17:a:z35			3 (4.2)		3 (0.9)
18:z35:-	1 (0.6)		2 (2.8)		3 (0.9)
23:-:6	1 (0.6)		2 (2.8)		3 (0.9)
Newport			3 (4.2)		3 (0.9)
Agona		2 (2.2)			2 (0.6)
II Lethe	1 (0.6)		1 (1.4)		2 (0.6)
Muenster	1 (0.6)	1 (1.1)			2 (0.6)
45:a:z35				1 (7.1)	1 (0.3)
50:-:-				1 (7.1)	1 (0.3)
52:-:1,5,7				1 (7.1)	1 (0.3)
60:r:-	1 (0.6)				1 (0.3)
Infantis	1 (0.6)				1 (0.3)
Litchfield			1 (1.4)		1 (0.3)
Newlands	1 (0.6)				1 (0.3)
Newrochelle	1 (0.6)				1 (0.3)
Paratyphi B	1 (0.6)				1 (0.3)
Senftenberg	1 (0.6)				1 (0.3)
Unidentified		1 (1.1)			1 (0.3)
Total	159	90	71	14	334

### Multilocus sequence typing (MLST) analysis

In total, 51 different ST patterns were identified among the 334 *Salmonella* isolates, and ST198 (13.2%, 44/334), ST11 (10.5%, 35/334), ST19 (7.8%, 26/334), ST451 (7.2%, 24/334), ST34 (6.3%, 21/334), and ST155 (6.0%, 20/334) were the most common STs (Table 2; Table S3). The top six (11.8%, 6/51) STs were accounted for 50.9% (170/334) of the isolates. Notably, although ST198 contained the largest number of isolates, these strains belonged to only two serovars, Kentucky (75.0%, 33/44) and Takoradi (25.0%, 11/44). Similarly, ST19 and ST34 were either a Typhimurium or a Typhimurium monophasic variant. In addition, ST11, with the second highest number of isolates, was mostly attributed to Enteritidis (82.9%, 29/35), whereas ST451, with the fourth highest number of isolates, was mostly attributed to Pomona (95.8%, 23/24). However, all strains determined to be *Salmonella* Give were classified as ST155. Similar to the relationship between serovars and season, no correlation was found between the distribution of STs and season.

**TABLE 2:** Sequence types of *Salmonella* isolated from pets in Chongqing, China

Sequence type	Dogs (%)	Cats (%)	Turtles (%)	Lizards (%)	Total (%)
ST198	25 (15.7)	19 (21.1)			44 (13.2)
ST11	19 (11.9)	16 (17.8)			35 (10.5)
ST19	17 (10.7)	9 (10.0)			26 (7.8)
ST451	1 (0.6)	4 (4.4)	19 (26.8)		24 (7.2)
ST34	14 (8.8)	7 (7.8)			21 (6.3)
ST155	13 (8.2)	7 (7.8)			20 (6.0)
ST413	14 (8.8)	1 (1.1)			15 (4.5)
ST50	4 (2.5)	5 (5.6)	4 (5.6)		13 (3.9)
ST469	8 (5.0)	4 (4.4)			12 (3.6)
ST617		1 (1.1)	3 (4.2)	7 (50.0)	11 (3.3)
ST40	6 (3.8)	4 (4.4)			10 (3.0)
ST358	7 (4.4)	1 (1.1)	1 (1.4)		9 (2.7)
ST2889			8 (11.3)		8 (2.4)
ST29	5 (3.1)	1 (1.1)	1 (1.4)		7 (2.1)
ST45			7 (9.9)		7 (2.1)
ST17	3 (1.9)	3 (3.3)			6 (1.8)
ST1306	4 (2.5)		1 (1.4)		5 (1.5)
ST343	1 (0.6)		3 (4.2)		4 (1.2)
ST434			4 (5.6)		4 (1.2)
ST1593	1 (0.6)	3 (3.3)			4 (1.2)
ST2259	1 (0.6)			3 (21.4)	4 (1.2)
ST20	2 (1.3)	1 (1.1)			3 (0.9)
ST83			3 (4.2)		3 (0.9)
ST287			3 (4.2)		3 (0.9)
ST2924	1 (0.6)		2 (2.8)		3 (0.9)
ST3918			3 (4.2)		3 (0.9)
ST13		2 (2.2)			2 (0.6)
ST27	2 (1.3)				2 (0.6)
ST166			2 (2.8)		2 (0.6)
ST314	2 (1.3)				2 (0.6)
ST2820			2 (2.8)		2 (0.6)
ST14	1 (0.6)				1 (0.3)
ST18			1 (1.4)		1 (0.3)
ST32	1 (0.6)				1 (0.3)
ST49	1 (0.6)				1 (0.3)
ST64	1 (0.6)				1 (0.3)
ST214			1 (1.4)		1 (0.3)
ST226			1 (1.4)		1 (0.3)
ST321		1 (1.1)			1 (0.3)
ST440			1 (1.4)		1 (0.3)
ST463	1 (0.6)				1 (0.3)
ST684	1 (0.6)				1 (0.3)
ST808				1 (7.1)	1 (0.3)
ST1197	1 (0.6)				1 (0.3)
ST1300				1 (7.1)	1 (0.3)
ST1499		1 (1.1)			1 (0.3)
ST2197				1 (7.1)	1 (0.3)
ST3038			1 (1.4)		1 (0.3)
ST4007				1 (7.1)	1 (0.3)
ST5494	1 (0.6)				1 (0.3)
ST7352	1 (0.6)				1 (0.3)

The strains derived from dogs, cats, turtles, and lizards belonged to 30, 19, 21, and 6 different STs, respectively. The three most common STs among the dog- and cat-derived isolates were ST198 (dog-derived: 15.7%, 25/159; cat-derived: 21.1%, 19/90), ST11 (dog-derived: 11.9%, 19/159; cat-derived: 17.8%, 16/90), and ST19 (dog-derived: 10.7%, 17/159; cat-derived: 10.0%, 9/90). However, the most prevalent STs of the turtle- and lizard-associated isolates were ST451 and ST617, respectively, which accounted for 26.8% (19/71) and 50.0% (7/14) of the isolates, respectively.

### Antimicrobial susceptibility profiles of *Salmonella* isolates

Among the 334 *Salmonella* isolates, 6 (8.5%, 6/334) from turtles and 1 (0.3%, 1/334) from a dog were susceptible to all tested antimicrobials, whereas the others showed resistance to at least one compound. High rates of resistance to sulfisoxazole (88.0%, 294/334), ampicillin (57.5%, 192/334), doxycycline (52.7%, 176/334), and tetracycline (51.5%, 172/334) were detected among the isolates. However, isolates were less often resistant to cefepime (3.6%, 12/334), cefoxitin (5.1%, 17/334), or gatifloxacin (8.7%, 29/334) (Table 3). Interestingly, in general, the resistance rate of isolates from reptiles seemed to be lower than that of isolates from dogs and cats to most antimicrobials. For instance, lizard-associated isolates were more resistant to sulfisoxazole (92.9%, 13/14), imipenem (57.1%, 8/14), and cefotaxime (42.9%, 6/14) and less resistant to cefazolin (14.3%, 2/14), ceftazidime (7.1%, 1/14), aztreonam (7.1%, 1/14), streptomycin (7.1%, 1/14), tetracycline (7.1%, 1/14), and doxycycline (7.1%, 1/14) but were susceptible to the other 19 antimicrobials.

**TABLE 3:** Antimicrobial resistance profiles of *Salmonella* isolated from dogs, cats, turtles, and lizards as determined by the disk diffusion method^*a*^

Antimicrobial classes	Antimicrobial	Dogs (%)	Cats (%)	Turtles (%	Lizards (%)	Total (%)
β-lactam	Ampicillin	108 (67.9)	68 (75.6)	16 (22.5)		192 (57.5)
	Cephalexin	60 (37.7)	41 (45.6)	8 (11.3)		109 (32.6)
	Cefazolin	71 (44.7)	52 (57.8)	7 (9.9)	2 (14.3)	132 (39.5)
	Cefoxitin	10 (6.3)	5 (5.6)	2 (2.8)		17 (5.1)
	Cefotaxime	70 (44.0)	47 (52.2)	29 (40.8)	6 (42.9)	152 (45.5)
	Ceftriaxone	44 (27.7)	32 (35.6)	3 (4.2)		79 (23.7)
	Ceftazidime	24 (15.1)	17 (18.9)	2 (2.8)	1 (7.1)	44 (13.2)
	Cefepime	5 (3.1)	6 (6.7)	1 (1.4)		12 (3.6)
	Imipenem	51 (32.1)	34 (37.8)	23 (32.4)	8 (57.1%)	116 (34.7)
	Aztreonam	42 (26.4)	30 (33.3)	2 (2.8)	1 (7.1%)	75 (22.5)
Amioglycosides	Streptomycin	74 (46.5)	40 (44.4)	6 (8.5)	1 (7.1%)	121 (36.2)
	Kanamycin	51 (32.1)	35 (38.9)	16 (22.5)		102 (30.5)
	Gentamicin	47 (29.6)	30 (33.3)	2 (2.8)		79 (23.7)
	Amikacin	27 (17.0)	23 (25.6)	4 (5.6)		54 (16.2)
Tetracyclines	Tetracycline	94 (59.1)	59 (65.6)	18 (25.4)	1 (7.1%)	172 (51.5)
	Doxycycline	95 (59.7)	61 (67.8)	19 (26.8)	1 (7.1%)	176 (52.7)
Amphenicols	Chloramphenicol	71 (44.7)	46 (51.1)	7 (9.9)		124 (37.1)
	Florfenicol	68 (42.8)	39 (43.3)	2 (2.8)		109 (32.6)
Quinolones	Nalidixic acid	60 (37.7)	44 (48.9)	10 (14.1)		114 (34.1)
	Norfloxacin	29 (18.2)	24 (26.7)			53 (15.9)
	Ciprofloxacin	41 (25.8)	33 (36.7)	10 (14.1)		84 (25.1)
	Enrofloxacin	44 (27.7)	31 (34.4)	3 (4.2)		78 (23.4)
	Ofloxacin	28 (17.6)	26 (28.9)			54 (16.2)
	Enoxacin	35 (22.0)	27 (30.0)			62 (18.6)
	Gatifloxacin	10 (6.3)	19 (21.1)			29 (8.7)
Sulfonamides	Trimethoprim	68 (42.8)	34 (37.8)	12 (16.9)		114 (34.1)
	Sulfisoxazole	141 (88.7)	81 (90.0)	59 (83.1)	13 (92.9)	294 (88.0)
	Trimethoprim-sulfamethoxazole	66 (41.5)	35 (38.9)	11 (15.5)		112 (33.5)
Total		159	90	71	14	334

^*a*^ The numbers in the table represent the number of resistant isolates and the percentage of strains isolated from different pets.

**Fig 1:**
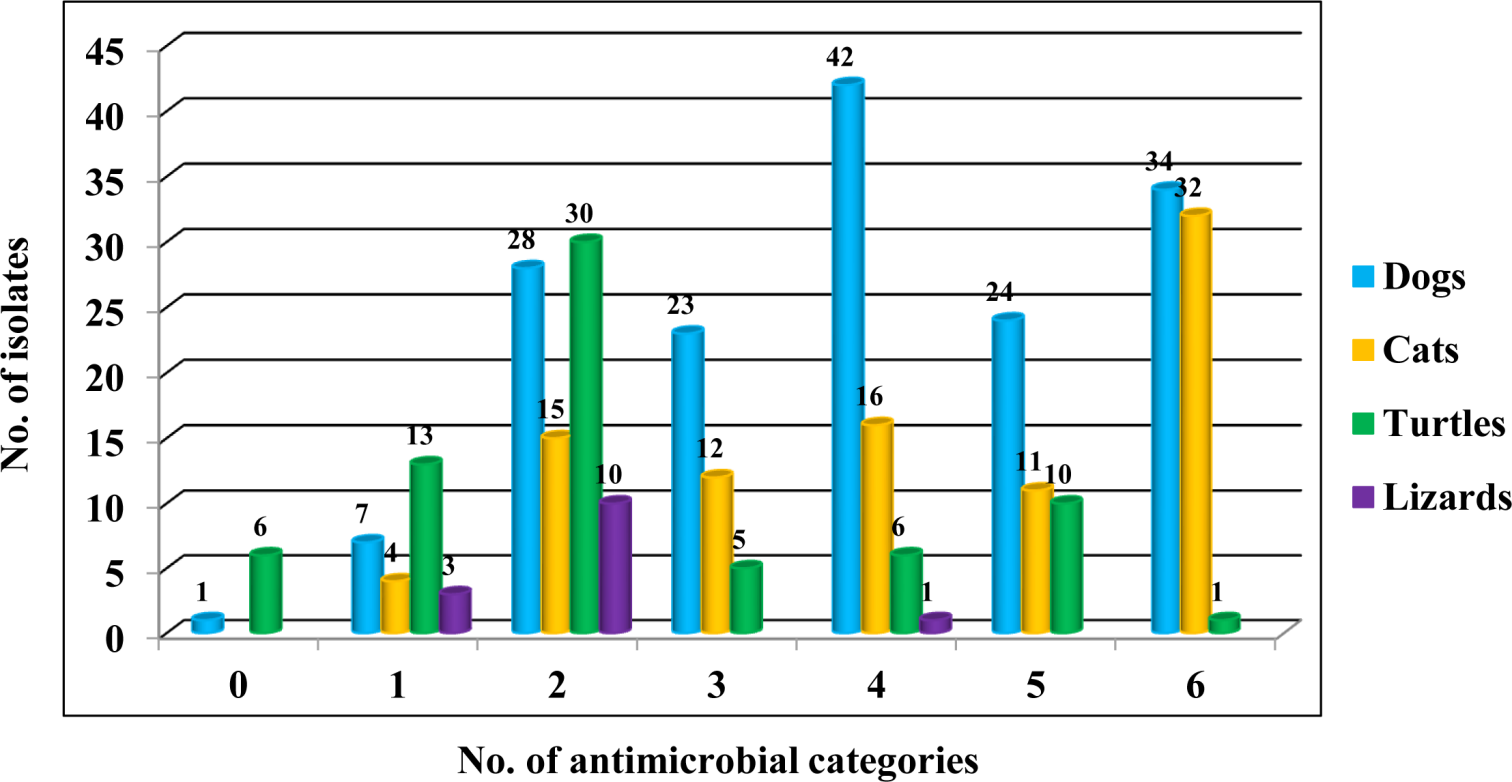
Multidrug-resistant *Salmonella* isolated from pet animals in Chongqing. The number of isolates resistant to different categories of antimicrobials is represented on the X axis. The number of isolates is indicated on the Y axis. Colors indicate different pets. Each of the histograms represents the number of isolates of each pet.

Among the 334 *Salmonella* isolates, 217 (65.0%, 217/334) were resistant to three or more classes of antimicrobials and were classified as MDR (Fig. 1). Similar to AMR, the prevalence of MDR in isolates obtained from reptiles was significantly lower than that in isolates acquired from dogs and cats (*P* < 0.01). However, there was no significant difference in the percentage of MDR between isolates from dogs and cats or from turtles and lizards. All strains belonging to serovars Kentucky, Saintpaul, Takoradi, and Abony exhibited MDR profiles, and most of the isolates belonging to serovars Typhimurium monophasic variant (79.5%, 31/39), Enteritidis (96.6%, 28/29), Give (75.0%, 15/20), Typhimurium (85.7%, 12/14), and Bovismorbificans (90.9%, 10/11) showed MDR phenotypes (Table 4). However, no isolate was considered to be multidrug-resistant among serovars 17:a:z35, 18:z35:-, 45:a:z35, 52:-:1,5,7, Cerro, Chester, Cotham, Essen, Infantis, Litchfield, Newport, and Senftenberg strains. From the perspective of STs, all strains of ST17, ST50, ST198, and ST358 exhibited MDR profiles, and most isolates belonging to ST34 (90.5%, 19/21), ST469 (83.3%, 10/12), ST11 (82.9%, 29/35), ST19 (80.8%, 21/26), ST40 (80.0%, 8/10), and ST155 (75.0%, 15/20) were multidrug-resistant (Table 5). In addition, strains assigned to 19 STs, including ST14, ST20, ST32, ST49, ST166, ST214, ST287, ST343, ST440, ST617, ST684, ST808, ST1300, ST1499, ST1593, ST2924, ST3038, ST3918, and ST4007, were resistant to fewer than two classes of antimicrobials.

**TABLE 4:** Distribution of multidrug-resistant *Salmonella* isolates among different serovars

Serovar	Dogs (%)	Cats (%)	Turtles (%)	Lizards (%)	Total (%)
Kentucky	22/22 (100.0)	12/12 (100.0)			34/34 (100.0)
Typhimurium monophasic variant	22/25 (88.0)	9/14 (64.3)			31/39 (79.5)
Enteritidis	17/18 (94.4)	11/11 (100.0)			28/29 (96.6)
Give	9/13 (69.2)	6/7 (85.7)			15/20 (75.0)
Saintpaul	6/6 (100.0)	5/5 (100.0)	4/4 (100.0)		15/15 (100.0)
Typhimurium	8/10 (80.0)	4/4 (100.0)			12/14 (85.7)
Takoradi	4/4 (100.0)	7/7 (100.0)			11/11 (100.0)
Bovismorbificans	8/8 (100.0)	1/2 (50.0)	1/1 (100.0)		10/11 (90.9)
Rissen	6/8 (75.0)	2/3 (66.7)			8/11 (72.7)
Derby	4/6 (66.7)	4/4 (100.0)			8/10 (80.0)
Pomona	0/1	3/4 (75.0)	3/18 (16.7)		6/23 (26.1)
Abony	2/2 (100.0)	3/3 (100.0)	1/1 (100.0)		6/6 (100.0)
23:z:-			5/6 (83.3)		5/6 (83.3)
61:z:z44	4/4 (100.0)		0/5		4/9 (44.4)
Manhattan			3/5 (60.0)		3/5 (60.0)
Stanley	4/5 (80.0)	0/1	2/2 (100.0)		2/8 (25.0)
Agona		2/2 (100.0)			2/2 (100.0)
Bokanjac			2/5 (40.0)	0/1	2/6 (33.3)
Mbandaka	1/13 (7.7)	0/1			1/14 (7.1)
Kottbus			1/6 (16.7)	0/1	1/7 (14.3)
23:-:6	1/1 (100.0)		0/2		1/3 (33.3)
II Lethe	1/1 (100.0)		0/1		1/2 (50.0)
Muenster	0/1	1/1 (100.0)			1/2 (50.0)
50:-:-				1/1 (100.0)	1/1 (100.0)
60:r:-	1/1 (100.0)				1/1 (100.0)
Newlands	1/1 (100.0)				1/1 (100.0)
Newrochelle	1/1 (100.0)				1/1 (100.0)
Paratyphi B	1/1 (100.0)				1/1 (100.0)
Unidentified		1/1 (100.0)			1/1 (100.0)
Cotham		0/1	0/3	0/9	0/13
Chester	0/2		0/3		0/5
Essen	0/1	0/4			0/5
Cerro	0/1	0/3			0/4
17:a:z35			0/3		0/3
18:z35:-	0/1		0/2		0/3
Newport			0/3		0/3
45:a:z35				0/1	0/1
52:-:1,5,7				0/1	0/1
Infantis	0/1				0/1
Litchfield			0/1		0/1
Senftenberg	0/1				0/1
Total	123/159 (77.4)	71/90 (78.9)	22/71 (31.0)	1/14 (7.1)	217/334 (65.0)

**TABLE 5:** Distribution of multidrug-resistant *Salmonella* isolates with different sequence types

Sequence type	Dogs (%)	Cats (%)	Turtles (%)	Lizards (%)	Total (%)
ST198	25/25 (100.0)	19/19 (100.0)			44/44 (100.0)
ST11	17/19 (89.5)	12/16 (75.0)			29/35 (82.9)
ST19	14/17 (82.4)	7/9 (77.8)			21/26 (80.8)
ST34	14/14 (100.0)	5/7 (71.4)			19/21 (90.5)
ST155	9/13 (69.2)	6/7 (85.7)			15/20 (75.0)
ST50	4/4 (100.0)	5/5 (100.0)	4/4 (100.0)		13/13 (100.0)
ST469	7/8 (87.5)	3/4 (75.0)			10/12 (83.3)
ST358	7/7 (100.0)	1/1 (100.0)	1/1 (100.0)		9/9 (100.0)
ST40	4/6 (66.7)	4/4 (100.0)			8/10 (80.0)
ST451	0/1	3/4 (75.0)	4/19 (21.1)		7/24 (29.2)
ST17	3/3 (100.0)	3/3 (100.0)			6/6 (100.0)
ST2889			5/8 (62.5)		5/8 (62.5)
ST29	4/5 (80.0)	0/1	1/1 (100.0)		5/7 (71.4)
ST1306	4/4 (100.0)		0/1		4/5 (80.0)
ST83			2/3 (66.7)		2/3 (66.7)
ST13		2/2 (100.0)			2/2 (100.0)
ST27	2/2 (100.0)				2/2 (100.0)
ST314	2/2 (100.0)				2/2 (100.0)
ST413	1/14 (7.1)	0/1			1/15 (6.7)
ST45			1/7 (14.3)		1/7 (14.3)
ST434			1/4 (25.0)		1/4 (25.0)
ST2259	1/1 (100.0)			0/3	1/4 (25.0)
ST2820			1/2 (50.0)		1/2 (50.0)
ST18			1/1 (100.0)		1/1 (100.0)
ST64	1/1 (100.0)				1/1 (100.0)
ST226			1/1 (100.0)		1/1 (100.0)
ST321		1/1 (100.0)			1/1 (100.0)
ST463	1/1 (100.0)				1/1 (100.0)
ST1197	1/1 (100.0)				1/1 (100.0)
ST2197				1/1 (100.0)	1/1 (100.0)
ST5494	1/1 (100.0)				1/1 (100.0)
ST7352	1/1 (100.0)				1/1 (100.0)
ST617		0/1	0/3	0/7	0/11
ST343	0/1		0/3		0/4
ST1593	0/1	0/3			0/4
ST20	0/2	0/1			0/3
ST287			0/3		0/3
ST2924	0/1		0/2		0/3
ST3918			0/3		0/3
ST166			0/2		0/2
ST14	0/1				0/1
ST32	0/1				0/1
ST49	0/1				0/1
ST214			0/1		0/1
ST440			0/1		0/1
ST684	0/1				0/1
ST808				0/1	0/1
ST1300				0/1	0/1
ST1499		0/1			0/1
ST3038			0/1		0/1
ST4007				0/1	0/1
Total	123/159 (77.4)	71/90 (78.9)	22/71 (31.0)	1/14 (7.1)	217/334 (65.0)

### Distribution of β-lactamase genes and ESBL genes in *Salmonella* isolates

The prevalence of β-lactamase genes in *Salmonella* isolates was 59.6% (199/334), and of these isolates, 108 (67.9%, 108/159) were obtained from dogs, 65 (72.2%, 72/90) from cats, 25 (35.2%, 25/71) from turtles, and 1 (7.1%, 1/14) from a lizard (Table S4). The most prevalent β-lactamase gene was *bla*_TEM_ (55.4%, 185/334), followed by *bla*_CTX-M_ (19.8%, 66/334) and *bla*_OXA_ (3.0%, 10/334), and no other β-lactamase gene was detected. The *bla*_TEM_ gene was identified in all four types of pet-derived *Salmonella* strains, and its genotypes were TEM-1 (60.5%, 112/185), TEM-116 (22.2%, 41/185), TEM-171 (0.5%, 1/185), TEM-201 (15.7%, 29/185), and TEM-244 (1.1%, 2/185) (Table 6). The alleles of 66 *bla*_CTX-M_ genes that existed in *Salmonella* originating from dogs, cats, and turtles were CTX-M-14 (3.6%, 12/334), CTX-M-14b (0.9%, 3/334), CTX-M-55 (14.4%, 48/334), and CTX-M-65 (0.9%, 3/334). Ten *bla*_OXA-10_-harboring strains were detected, five of which were isolated from dogs, four from cats, and one from a turtle. The isolates carried one to three *bla* genes (Table S4). The most prevalent genotype was TEM-1 (21.9%, 73/334), followed by TEM-116 (11.7%, 39/334), TEM-1 +CTX M-55 (9.9%, 33/334), TEM-201 (5.1%, 17/334), and TEM-201 +CTX M-55 (3.0%, 10/334).

**TABLE 6:** Prevalence of β-lactamase genes in *Salmonella* isolates originating from different pets^*a*^

Genotype of β-lactamase gene		Dogs (%) (*n* = 159)	Cats (%) (*n* = 90)	Turtles (%) (*n* = 71)	Lizards (%) (*n* = 14)	Total (%) (*n* = 334)
TEM	TEM-1	65 (40.9)	42 (46.7)	5 (7.0)		112 (33.5)
	TEM-116	15 (9.4)	5 (5.6)	20 (28.2)	1 (7.1)	41 (12.3)
	TEM-171	1 (0.6)				1 (0.3)
	TEM-201	18 (11.3)	11 (12.2)			29 (8.7)
	TEM-244	1 (0.6)	1 (1.1)			2 (0.6)
CTX-M	CTX-M-14	9 (5.7)	3 (3.3)			12 (3.6)
	CTX-M-14b	3 (1.9)				3 (0.9)
	CTX-M-55	22 (13.8)	25 (27.8)	1 (1.4)		48 (14.4)
	CTX-M-65	2 (1.3)	1 (1.1)			3 (0.9)
OXA	OXA-10	5 (3.1)	4 (4.4)	1 (1.4)		10 (3.0)

^*a*^ Genes marked in green are ESBL-producing genes.

The ESBL genes included *bla*_CTX-M_, *bla*_SHV_, and *bla*_TEM_, not *bla*_TEM-1_ or *bla*_TEM-2_. In this study, we identified 127 (38.0%, 127/334) ESBL-producing strains, including 65 dog-associated (40.9%, 65/159), 40 cat-associated (44.4%, 44/90), 21 turtle-associated (29.6%, 21/71), and one lizard-associated (7.1%, 1/14) isolates. Of the 127 ESBL-producing isolates, 109 (85.8%) were found to be resistant to extended-spectrum cephalosporins and monobactam, including 90.8% (59/65) dog-associated strains, 92.5% (37/40) cat-associated strains, and 59.1% (13/22) turtle-associated strains. Tables 7 and 8 showed that more than half of the isolates belonging to serovars Abony (100.0%, 6/6), Takoradi (100.0%, 11/11), Kentucky (94.1%, 32/34), 23:-:6 (66.7%, 2/3), Rissen (63.6%, 7/11), Kottbus (57.1%, 4/7), and Typhimurium (50.0%, 7/14), as well as 5 STs, including ST17 (100.0%, 6/6), ST198 (95.5%, 42/44), ST45 (71.4%, 5/7), ST469 (58.3%, 7/12), and ST19 (53.8%, 14/26), were ESBL-positive. However, there was no isolate-harbored ESBL genes among serovars 17:a:z35, 18:z35:-, 23:z:-, 45:a:z35, 50:-:-, 52:-:1,5,7, 60:r:-, II Lethe, Cerro, Chester, Essen, Infantis, Litchfield, Manhattan, and Newlands and 24 STs, including ST18, ST20, ST32, ST49, ST64, ST83, ST166, ST214, ST343, ST440, ST617, ST684, ST808, ST1197, ST1300, ST1499, ST1593, ST2197, ST2820, ST2924, ST3918, ST4007, ST5494, and ST7352.

**TABLE 7:** Distribution of ESBL-producing *Salmonella* isolates among different serovars

Serovar	Dogs (%)	Cats (%)	Turtles (%)	Lizards (%)	Total (%)
23:-:6			2/2 (100.0)		2/3 (66.7)
61:z:z44	2/4 (50.0)		2/5 (40.0)		4/9 (44.4)
Abony	2/2 (100.0)	3/3 (100.0)	1/1 (100.0)		6/6 (100.0)
Agona		2/2 (100.0)			2/2 (100.0)
Bokanjac			2/5 (40.0)		2/6 (33.3)
Bovismorbificans			1/1 (100.0)		1/11 (9.1)
Cotham				1/9 (11.1)	1/13 (7.7)
Derby	2/6 (33.3)				2/10 (20.0)
Enteritidis	2/18 (11.1)	3/11 (27.2)			5/29 (17.2)
Give	2/13 (15.4)				2/20 (10.0)
Kentucky	20/22 (90.9)	12/12 (100.0)			32/34 (94.1)
Kottbus			4/6 (66.7)		4/7 (57.1)
Mbandaka	1/13 (7.7)				1/14 (7.1)
Muenster		1/1 (100.0)			1/2 (50.0)
Newport			1/3 (33.3)		1/3 (33.3)
Newrochelle	1/1 (100.0)				1/1 (100.0)
Paratyphi B	1/1 (100.0)				1/1 (100.0)
Pomona			6/18 (33.3)		6/23 (26.1)
Rissen	5/8 (62.5)	2/3 (66.7)			7/11 (63.6)
Saintpaul	5/6 (83.3)	1/5 (20.0)	1/4 (25.0)		7/15 (46.7)
Senftenberg	1/1 (100.0)				1/1 (100.0)
Stanley			1/2 (50.0)		1/8 (12.5)
Takoradi	4/4 (100.0)	7/7 (100.0)			11/11 (100.0)
Typhimurium	4/10 (40.0)	3/4 (75.0)			7/14 (50.0)
Typhimurium monophasic variant	13/25 (52.0)	6/14 (42.9)			19/39 (48.7)
Total	65/159 (40.9)	40/90 (44.4)	21/71 (29.6)	1/14 (7.1)	127/334 (38.0)

**TABLE 8:** Distribution of ESBL-producing *Salmonella* isolates in different sequence types

Sequence type	Dogs (%)	Cats (%)	Turtles (%)	Lizards (%)	Total (%)
ST11	2/19 (10.5)	3/16 (18.8)			5/35 (14.3)
ST13		2/2 (100.0)			2/2 (100.0)
ST14	1/1 (100.0)				1/1 (100.0)
ST17	3/3 (100.0)	3/3 (100.0)			6/6 (100.0)
ST19	9/17 (52.9)	5/9 (55.6)			14/26 (53.8)
ST27	2/2 (100.0)				2/2 (100.0)
ST29			1/1 (100.0)		1/7 (14.3)
ST34	7/14 (50.0)	3/7 (42.9)			10/21 (47.6)
ST40	2/6 (33.3)				2/10 (20.0)
ST45			5/7 (71.4)		5/7 (71.4)
ST50	3/4 (75.0)	1/5 (20.0)	1/4 (25.0)		5/13 (38.5)
ST155	2/13 (15.4)				2/20 (10.0)
ST198	23/25 (92.0)	19/19 (100.0)			42/44 (95.5)
ST226			1/1 (100.0)		1/1 (100.0)
ST287			1/3 (33.3)		1/3 (33.3)
ST314	2/2 (100.0)				2/2 (100.0)
ST321		1/1 (100.0)			1/1 (100.0)
ST358			1/1 (100.0)		1/9 (11.1)
ST413	2/14 (14.3)				2/15 (13.3)
ST434			1/4 (25.0)		1/4 (25.0)
ST451			7/19 (36.8)		7/24 (29.2)
ST463	1/1 (100.0)				1/1 (100.0)
ST469	4/8 (50.0)	3/4 (75.0)			7/12 (58.3)
ST1306	2/4 (50.0)				2/5 (40.0)
ST2259				1/3 (33.3)	1/4 (25.0)
ST2889			2/8 (25.0)		2/8 (25.0)
ST3038			1/1 (100.0)		1/1 (100.0)
Total	65/159 (40.9)	40/90 (44.4)	21/71 (29.6)	1/14 (7.1)	127/334 (38.0)

### Characterization of PMQR and QRDR mutations among *Salmonella* isolates

One hundred and fifty strains isolated from dogs (47.2%, 75/159), cats (60.0%, 54/90), and turtles (29.6%, 21/71) were resistant to at least one tested quinolone, and most of the quinolone-resistant strains had a mutation(s) in *gyrA* and/or *parC* (88.7%, 133/150). Some of these strains harbored 1–4 PMQR gene(s) (39.7%, 60/151). However, QRDR point mutation(s) and PMQR gene(s) were detected in 256 (76.6%) and 114 (34.1%) *Salmonella* isolates, respectively, regardless of whether they were resistant to the quinolones tested. Seventeen mutation patterns were discovered in the *gyrA* and/or *parC* gene after sequencing (Table 9). The most frequent mutation was T57S in *parC* (47.3%, 158/334), followed by a combination of mutations at two sites in *gyrA* (S83F + D87N) and in *parC* (T57S + S80I) (11.7%, 39/334) and a mutation in *gyrA* (D87Y) (6.0%, 20/334).

**TABLE 9:** Distribution of QRDR mutations in pet-associated *Salmonella* isolates

QRDR mutation(s)		Dogs (%) (*n* = 159)	Cats (%) (*n* = 90)	Turtles (%) (*n* = 71)	Lizards (%) (*n* = 14)	Total (%) (*n* = 334)
gyrA	parC					
S83F		2 (1.3)				2 (0.6)
S83Y			3 (3.3)			3 (0.9)
D87Y		15 (9.4)	5 (5.6)			20 (6.0)
D87L			1 (1.1)			1 (0.3)
D87N		1 (0.6)				1 (0.3)
D87G		2 (1.3)	2 (2.2)			4 (1.2)
S83F, D87G			1 (1.1)			1 (0.3)
	T57S	65 (40.9)	24 (26.7)	60 (84.5)	9 (64.3)	158 (47.3)
	T57S, S80I		1 (1.1)			1 (0.3)
S83F	T57S	6 (3.8)	1 (1.1)			7 (2.1)
S83Y	T57S		2 (2.2)	4 (5.6)		6 (1.8)
D87Y	T57S		2 (2.2)			2 (0.6)
S83F, D87N	T57S	3 (1.9)				3 (0.9)
S83F, D87G	T57S, S80I	3 (1.9)				3 (0.9)
S83F, D87G	T57S, S80R	1 (0.6)				1 (0.3)
S83F, D87N	T57S, S80I	20 (12.6)	19 (21.1)			39 (11.7)
S83F, D87N	T57S, S80R	1 (0.6)	3 (3.3)			4 (1.2)
Total		119 (74.8)	64 (71.1)	64 (90.1)	9 (64.3)	256 (76.6)

Six PMQR genes were identified in 114 PMQR-positive isolates, namely, *qnrA* (14.0%, 16/114), *qnrB* (12.3%, 14/114), *qnrD* (2.6%, 3/114), *qnrS* (55.3%, 63/114), *aac(6')-Ib-cr* (48.2%, 55/114), and *oqxB* (4.4%, 5/114) (Table 10). *QnrC*, *qnrVC*, *oqxA*, and *qepA* were not detected in any of the isolates. The genotypes of *qnrA*, *qnrD*, and *oqxB* were *qnrA1*, *qnrD1*, and *oqxB10*, respectively. Additionally, *qnrB* represented *qnrB4* (0.9%, 1/114) and *qnrB6* (11.4%, 13/114). Moreover, *qnrS* represented *qnrS2* (5.3%, 6/114) and *qnrS10* (50.0%, 57/114). Similar to the case for β-lactamase gene-producing isolates, there were a total of 14 combinations of PMQR genes in PMQR-positive strains (Table S5). The dominant forms were two single PMQR genes, *qnrS10* and *aac(6')-Ib-cr*, which represented 56 (49.1%) and 19 (16.7%) of the PMQR-positive isolates, respectively. Two combinations, *qnrA1 +aac(6')-Ib-cr* (13.2%, 15/114) and *qnrB6 +aac(6')-Ib-cr* (8.8%, 10/114), were also prevalent among PMQR-positive strains. Incidentally, PMQR genes were absent from all lizard-derived isolates that were not resistant to the tested quinolones. Of the 114 PMQR-producing isolates, 58 (50.8%) were found to be resistant to the tested quinolones, including 44.1% (26/59) dog-associated strains, 61.8% (21/34) cat-associated strains, and 52.4% (11/21) turtle-associated strains.

**TABLE 10:** Prevalence of PMQR genes in *Salmonella* isolates originating from pets

Genotype of PMQR genes		Dogs (%) (*n* = 59)	Cats (%) (*n* = 34)	Turtles (%) (*n* = 21)	Total (%) (*n* = 114)
*qnrA*	*qnrA1*	4 (6.8)	6 (17.6)	6 (28.6)	16 (14.0)
*qnrB*	*qnrB4*			1 (4.8)	1 (0.9)
	*qnrB6*	5 (8.5)	1 (2.9)	7 (33.3)	13 (11.4)
*qnrD*	*qnrD1*	1 (1.7)	1 (2.9)	1 (4.8)	3 (2.6)
*qnrS*	*qnrS2*	3 (5.1)	3 (8.8)		6 (5.3)
	*qnrS10*	32 (54.2)	20 (58.8)	5 (23.8)	57 (50.0)
*aac(6')-Ib-cr*		26 (44.1)	13 (38.2)	16 (76.2)	55 (48.2)
*oqxB*	*oqxB10*	4 (6.8)	1 (2.9)		5 (4.4)

### Tetracycline resistance genotypes of *Salmonella* isolates

A total of 176 (52.7%) *Salmonella* isolates carried tetracycline resistance genes, although nine strains harboring *tet* genes did not show resistance to tetracycline and/or doxycycline (Table S6). The prevalence of tetracycline resistance genes in organisms isolated from dogs, cats, turtles, and lizards was 61.0% (97/159), 66.7% (60/90), 25.4% (18/71), and 7.1% (1/14), respectively (Table S7). Among the tetracycline resistance genes analyzed, *tet*(A) was the most prevalent, present in 39.5% (132/334) of the isolates, followed by *tet*(B) (8.1%, 27/334), *tet*(M) (7.8%, 26/334), *tet*(D) (5.4%, 18/334), *tet*(J) (3.3%, 11/334), and *tet*(C) (1.8%, 6/334), but no *tet*(E), *tet*(G), *tet*(S), *tet*(X), *tetP*(A), *tet*(X1), *tet*(X2), *tet*(X3), *tet*(X4) or *tet*(X5) was detected (Table 11). From the origins of the isolates, all six identified tetracycline resistance genes could be detected in dog-derived isolates; five *tet* genes except for *tet*(C) could be detected in cat-derived isolates; *tet*(A), *tet*(B), and *tet*(D) were amplified in turtle-derived isolates; and only one isolate obtained from a lizard carried *tet*(B) gene.

**TABLE 11:** Prevalence of tetracycline resistance genes in *Salmonella* isolates obtained from pets

Genotype of tetracycline resistance gene	Dogs (%) (*n* = 159)	Cats (%) (*n* = 90)	Turtles (%) (*n* = 71)	Lizards (%) (*n* = 14)	Total (%) (*n* = 334)
*tet*(A)	73 (45.9)	48 (53.3)	11 (15.5)		132 (39.5)
*tet*(B)	15 (9.4)	8 (8.9)	3 (4.2)	1 (7.1)	27 (8.1)
*tet*(C)	6 (3.8)				6 (1.8)
*tet*(D)	8 (5.0)	5 (5.6)	5 (7.0)		18 (5.4)
*tet*(J)	8 (5.0)	3 (3.3)			11 (3.3)
*tet*(M)	20 (12.6)	6 (6.7)			26 (7.8)

There were 98, 19, 17, 1, and 2 isolates each carrying only *tet*(A), *tet*(B), *tet*(D), *tet*(J), and *tet*(M), respectively. The remaining 39 tetracycline resistance gene-positive strains harbored 2–3 tetracycline resistance genes, with the most common co-occurrence being *tet*(A) +*tet*(M) (6.9%, 23/334), followed by *tet*(A) +*tet*(B) (1.2%, 4/334) and *tet*(A) +*tet*(C) +*tet*(J) (1.2%, 4/334). The remaining five combinations each represented 1–2 strains (Table S7).

### Occurrence of sulfonamide resistance genes in *Salmonella* isolates

Sulfonamide resistance genes (82.9%, 277/334) were detected in most *Salmonella* isolates that were resistant to the tested sulfonamides, although some isolates (7.8%, 26/334) susceptible to the tested sulfonamides also harbored sulfonamide resistance genes (Table S8). *sul1* was the most prevalent sulfonamide resistance gene among the isolated strains, with 84.4% (282/334) of the strains carrying this gene, followed by *sul2* (31.1%, 104/334). The number of isolates carrying *sul3* (4.2%, 14/334) was the lowest, although all *sul3*-positive isolates were resistant to sulfonamides (Table 12). There were six genotype combinations of sulfonamide resistance genes (Table S9). A total of 212 isolates (63.5%) carried only one sulfonamide resistance gene, with the highest occurrence of *sul1* (59.0%, 197/334), followed by *sul2* (4.2%, 14/334) and *sul3* (0.3%, 1/334). In addition, 90 isolates simultaneously harbored two or three sulfonamide resistance genes, in the forms of *sul1 + sul2* (23.1%, 77/334), *sul2 + sul3* (1.5%, 5/334), and *sul1 + sul2 + sul3* (2.4%, 8/334).

**TABLE 12:** Prevalence of sulfonamide resistance genes in *Salmonella* isolates obtained from pets

Genotype of sulfonamide resistance genes	Dogs (%) (*n* = 159)	Cats (%) (*n* = 90)	Turtles (%) (*n* = 71)	Lizards (%) (*n* = 14)	Total (%) (*n* = 334)
*Sul1*	133 (83.6)	79 (52.2)	57 (80.3)	13 (92.9)	282 (84.4)
*Sul2*	68 (42.8)	32 (35.6)	3 (4.2)	1 (7.1)	104 (31.1)
*Sul3*	9 (5.7)	4 (4.4)	1 (1.4)	0	14 (4.2)

### Conjugation with isolates harboring four types of resistance genes

A total of 86 isolates (25.7%, 86/344) simultaneously carried four types of detected resistance genes (β-lactamase genes, PMQR genes, and tetracycline and sulfonamide resistance genes), including 48 isolates (55.8%, 48/86) from dogs, 30 (34.9%, 30/86) from cats, and 8 (9.3%, 8/86) from turtles. These isolates were all multidrug-resistant. These isolates were distributed across 16 serovars (Table S10), among which 100% of serovar Saintpaul as well as more than 30% of serovars Abony (66.7%, 4/6), Bovismorbificans (63.6%, 7/11), Derby (60.0%, 6/10), Give (45.0%, 9/20), Typhimurium monophasic variant (43.6%, 17/39), Rissen (36.4%, 4/11), Typhimurium (35.7%, 5/14), and Kentucky (32.4%, 11/34) were all isolates of this type. In addition, these isolates belonged to 17 STs (Table S11): 100% (13/13) were ST50, 77.8% (7/9) were ST358, 60.0% (6/10) were ST40, 50.0% (13/26) were ST19, 45.0% (9/20) were ST155, 41.7% (5/12) were ST469, and 38.1% (8/21) were ST34.

Conjugation experiments demonstrated that at least two resistance genes were cotransferred from each of the 86 donor strains to the recipient bacterium *Escherichia coli* J53 Az^R^ (Table S12). In general, except for *bla*_CTX-M-14b_, *bla*_CTX-M-171_, *qnrD1*, and *sul3*, the other 23 detected resistance genes could be transferred to the recipient bacteria from the donors. In 39 strains (45.3%), all four types of resistance genes could be transferred to the recipients, of which 28 isolates (71.8%) transferred all resistance genes, including 3 isolates transferring 7 genes, 10 isolates transferring 6 genes, 19 isolates transferring 5 genes, and 7 isolates transferring 4 genes. From the perspective of resistance genes, *bla*_TEM-244_ (1), *bla*_CTX-M-65_ (2), *qnrB4* (1), and *oqxB10* (2) could all be transferred from donor to recipient cells. The conjugation-mediated transfer of *bla*_CTX-M-55_ (94.1%, 16/17) and *sul1* (94.7%, 72/76) was also robust, with rates all exceeding 90%, whereas the conjugation-mediated transfer of *tet*(M) (20.8%, 5/24) and *qnrS10* (36.7%, 18/49) was relatively poor (Table S13).

## DISCUSSION

As one of the most important zoonotic pathogens, people often pay attention to *Salmonella* in food-producing animals or food of animal origin transmission chains (28, 46, 47) while less attention has been given to *Salmonella* originating from pets. However, *Salmonella* colonizing the intestines of pet animals can also be transmitted to humans, leading to the onset of illness that requires attention. For example, one study reported that *Salmonella* Virchow was transmitted from household dogs to an infant manifesting diarrhea (26). In addition, multistate outbreaks of human *Salmonella* infections have been linked to small turtles (18) and pet hedgehogs (20) in the United States. In practice, this situation may be severely underestimated.

The prevalence of *Salmonella* among dogs and cats was 4.4% and 3.7%, respectively, in our study, which concurred with previous studies in Xuzhou, China (7.08% in dogs and 2.31% in cats) but was lower than the prevalence in other developing countries, such as Ethiopia (48), Thailand (14, 30), and Libya (49). Moreover, in this study, we showed fecal shedding of *Salmonella* among dogs and cats, in which a significantly greater prevalence was recorded in diarrheal dogs (6.9%) and cats (7.2%) than in apparently healthy dogs and cats (4.0% and 3.1%). This higher prevalence of *Salmonella* in diarrheal dogs and cats was supported by previous findings in different parts of the globe (13, 15, 48). In our study, the isolation rate of *Salmonella* derived from reptiles (turtle: 81.6%; lizard: 66.7%) was not only much greater than that in similar reports from Zhang et al. (turtle: 24.4%) (45) and Wang et al. (turtle: 14.5%) (31) in China but also greater than that in reports from other countries, including Grenada, the West Indies (turtle: 14.7%) (50), Australia (turtle: 20%; lizard: 39.6%; snake: 50%) (51), Portugal (turtle: 20.9%; lizard: 51.9%; snake: 50%) (52), and Korea (turtle: 60%) (29). Eighty-five (78.7%) reptile-derived *Salmonella* strains were isolated from 108 rectal samples collected at 3 pet clinics and 14 pet shops, indicating the widespread presence of *Salmonella* in pet reptiles in Chongqing. As some reports (13, 48) have stated, the prevalence of *Salmonella* in pets varies in different regions and countries, probably because of differences in pet sanitary practices, feeding habits, public awareness about pet zoonosis, and the socioeconomic status of the owners. In addition, some factors, such as differences in the season of study, geographical areas, and isolation methods employed, might have also accounted for the observed differences. According to some reports, the proportion of children aged <5 years infected with *Salmonella* was greater than that of adults (18, 31, 53), and some outbreaks were associated with pet-associated *Salmonella* (18). Therefore, it is particularly important to avoid direct contact between children and reptiles, such as grasping reptiles with bare hands.

A high degree of serovar diversity was observed among *Salmonella* isolates in this study. Interestingly, the Typhimurium monophasic variant was the most common serovar among the *Salmonella* isolates identified from both dogs and cats in our study, which was not common in previous studies. The *Salmonella* Typhimurium monophasic variant is prevalent mainly in humans, food, water, and animals and is strongly associated with the swine food chain (53–55). However, an outbreak of salmonellosis in a family in central Italy affected two children and involved their three dogs as carriers; phenotypic and genotypic analyses revealed clear transmission of the pathogen identified as a *Salmonella* Typhimurium monophasic variant between the human and nonhuman members of the family and the possibility of transmission from a dog to a human (56). Therefore, the prevalence of the *Salmonella* Typhimurium monophasic variant in pets and the risk of transmission from pets to humans cannot be ignored. The most common STs of the dog- and cat-derived isolates were ST198, ST11, and ST19. Among *Salmonella* isolates from pet dogs in Xuzhou, China, ST198, ST11, and ST34 were the most prevalent STs (57). However, 26 pet-derived *Salmonella* isolates were identified as ST10 (number: 22) and ST19 (number: 4) in Hangzhou, China (58). Therefore, the major STs of *Salmonella* isolated from dogs in Chongqing were roughly the same as those of *Salmonella* isolated from dogs in Xuzhou but different from those of *Salmonella* isolated from dogs and cats in Hangzhou.

Local pet hospital veterinarians reported common usage of ampicillin, ceftiofur, cefotaxime, cefovir, meropenem, doxycycline, gentamicin, tilmicosin, tylosin, and azithromycin for pets. However, pet-derived *Salmonella* isolates exhibit widespread resistance to some antimicrobials that are prohibited or not commonly used in pet clinical practice, such as chloramphenicol, florfenicol, tetracycline, and sulfonamides. For example, sulfonamides are rarely used in pets because the use of sulfonamides in pets sometimes causes the development of urinary stones. It has been reported that *Salmonella* strains isolated from pets in many countries, including China, are strongly resistant to β-lactams, tetracyclines, and/or sulfonamides (13–15, 30, 48). There might be two major reasons for the severe resistance of these isolates to certain antimicrobials that are not uncommonly used and prohibited in pet clinical practice. First, due to the widespread use of antimicrobials in food-producing animals, drug-resistant *Salmonella* bacteria from food-producing animals can be disseminated into the external environment, and pets can be infected with these drug-resistant bacteria during outdoor activities (59). In addition, pet foods contain a large amount of food-producing animal-associated raw materials, which might be contaminated by drug-resistant *Salmonella* (12, 59). Contaminated foods might transmit drug-resistant *Salmonella* to pets. Compared with *Salmonella* from dogs and cats, *Salmonella* from reptiles was less resistant to antimicrobials and carried fewer resistance genes. This might be due to the limited contact between pet reptiles and the external environment, resulting in fewer opportunities for acquiring drug-resistant *Salmonella*. In addition, reptiles are not usually fed with feed. For example, lizards mainly feed on insects than feed, which reduces the chances of lizards being infected with drug-resistant *Salmonella* in pet food containing raw meat or meat products.

The presence of multidrug-resistant organisms, especially *Salmonella* isolated from dogs and cats, cannot be ignored. Many different sequence types and serotypes found in dogs and cats in this study were MDR, showing that MDR is not a genotypically limited phenomenon but is indicative of a more widespread phenomenon. Notably, the proportion of multidrug-resistant *Salmonella* isolates from reptile sources was relatively low. Furthermore, there was no significant difference in the proportion of multidrug-resistant bacteria among the serotypes or genotypes for many isolates, indicating that *Salmonella*’s acquisition of resistance was not closely related to their serovars or STs.

Among 199 β-lactamase gene-positive *Salmonella* isolates, 127 were ESBL-producing strains, of which 52.8% (67/127) carried the *bla*_CTX-M_ gene. In our previous research on *Salmonella* originating from food-producing animals, the proportion of ESBL-producing isolates was 24.8% (32/129), and only a small number of strains (6.2%, 8/129) carried the *bla*_CTX-M_ gene, all of which were isolated from pigs. The proportion of pet-derived *Salmonella* strains isolated in Chongqing, both those producing ESBLs and those carrying the *bla*_CTX-M_ gene, was much greater than that of food-producing animal-associated *Salmonella* strains producing ESBLs and carrying the *bla*_CTX-M_ gene. This result implied that the source of *Salmonella* from pets is different from that of *Salmonella* from food-producing animals in Chongqing.

The coexistence or cotransference of PMQR genes in ESBL-producing strains, especially in *bla*_CTX-M_-producing isolates, has been described previously (60–63). Their coexistence might promote the development of multidrug-resistant isolates under the selective pressure of quinolones and/or cephalosporins (60, 61, 64). However, among the pet-derived *Salmonella* isolates, 41.7% (53/127) of the ESBL-producing strains carried PMQR gene(s), but no correlation was found between the cotransfer of the ESBL gene and the PMQR gene. The reason was that the positive rate of the PMQR gene was greater in β-lactamase gene-positive and non-ESBL-producing strains than in ESBL-producing strains, but the difference was not statistically significant. In addition, the PMQR gene exhibited low levels of resistance in *Salmonella*, as previously reported (65), as only 50% of the PMQR-positive strains were resistant to quinolones.

Tetracycline and sulfonamide resistance genes were prevalent in isolated *Salmonella*, with positive rates of 52.9% and 90.5%, respectively. Although these two types of resistance genes have rarely been reported in pet-derived *Salmonella*, they seem to be very common in *Salmonella* isolates from other sources. For example, the abundances of *tet*(B) and *sul2* were 94.2% and 91.9%, respectively, in 363 *Salmonella* isolates from Guizhou Province, China (37). Zhu et al. reported that a total of 189 *Salmonella* isolates were recovered from the cecal contents of broilers, chicken carcasses, chicken meat after the cutting step, and frozen broiler chicken products during the slaughtering process at a slaughterhouse in Sichuan Province, China; the tetracycline resistance genes (*tet*(A), *tet*(B), *tet*(C), and *tet*(G)) and sulfonamide resistance genes (*sul1*, *sul2*, and *sul3*) were identified in 84 (85.7%) and 89 (97.8%) antibiotic-resistant isolates, respectively (66). Moreover, a study carried out in Bangladesh showed that 81.4% of the *Salmonella* isolated from broiler farms harbored *tet*(A), 19.8% harbored *tet*(B), and 10.47% harbored *tet*(C), and the prevalence of *sul1* was 37.2% (67). The above results indicated that both tetracycline and sulfonamide resistance genes might be prevalent worldwide. The widespread prevalence of these two types of drug-resistance genes in *Salmonella* from different sources has led to widespread high-level resistance to tetracyclines and sulfonamides.

The resistance rates of the *Salmonella* isolates to the measured aminoglycosides and amphenicols were 16%–37% and 32%–38%, respectively. Furthermore, we ask the following: what are the resistance genes for aminoglycosides and amphenicols carried by the isolated strains, and how about the prevalence of these resistance genes? In future research, we will continue to detect the resistance genes of both these antimicrobials.

Conjugation experiments demonstrated the cotransference of different types of resistance genes from donor *Salmonella* to the recipient bacterium *E. coli* J53 Az^R^. However, we do not know whether plasmid conjugation can occur between *Salmonella* isolates. Due to plasmid incompatibility, further research is needed to verify the transfer and frequency of these resistant plasmids between *Salmonella* isolates. In addition, the structure of plasmids carrying multiple resistance genes deserves further exploration.

Based on the medication status of pets and the antimicrobial resistance and resistance genes of *Salmonella* isolates obtained from pets in Chongqing, we believe that reducing the use of antimicrobials in food-producing animals might be the key to controlling the development of drug resistance among *Salmonella*. Fortunately, China launched a campaign to reduce the use of antimicrobials in 2021 (http://www.xmsyj.moa.gov.cn/gzdt/202110/t20211025_6380448.htm). This effort might dramatically reduce the prevalence of drug-resistant *Salmonella*. In addition, misuse and overuse of antimicrobials in pets might also be important reasons for the emergence of drug-resistant strains. The Chinese government has begun to standardize medication use in pets and has carried out corresponding legislative work (http://www.moa.gov.cn/govpublic/xmsyj/202009/t20200914_6352002.htm; http://www.moa.gov.cn/govpublic/xmsyj/202210/t20221012_6413082.htm). It is hoped that with the implementation of the above actions, the resistance of pet-derived *Salmonella* to antimicrobials may be controlled.

In summary, we first investigated the prevalence, serovar diversity, STs, and antimicrobial resistance of *Salmonella* strains isolated from pet rectal swabs in Chongqing, China. In addition, β-lactamase, QRDR, PMQR, tetracycline and sulfonamide resistance genes, and mutations in QRDRs among *Salmonella* isolates were examined. Our findings demonstrated the diversity of serovars and STs among *Salmonella* isolates. The isolates were widely resistant to antimicrobials, notably with a high proportion of multidrug-resistant strains, which highlights the potential direct or indirect transmission of multidrug-resistant *Salmonella* from pets to humans. Furthermore, resistance genes were widely prevalent in the isolates, and most of the resistance genes were spread horizontally between species.

## MATERIALS AND METHODS

### Sample collection

From September 2018 to May 2021, a total of 6,223 fresh pet rectal swabs, including 3,638 from dogs, 2,409 from cats, 87 from turtles, 40 from parrots, 21 from lizards, 12 from hedgehogs, 7 from pet rabbits, 6 from pet rats, 2 from foxes, and 1 from a snake, were collected at 50 pet clinics, 42 pet shops, 7 residential areas, and 4 plazas in Chongqing municipality, China (Table S1). All experimental protocols were approved by the Institutional Animal Ethics Committee of Southwest University (approval no. IACUC-20170420–02) and were performed accordingly.

A small sterile cotton-tipped swab (Haoheng, Nanchang, China) was inserted into the anus or cloaca and rotated, gently scraping the mucosa of the lower rectum. The swab was placed into a sterile capped tube containing 2 mL of sterile buffered peptone water (BPW) (Hopebio, Qingdao, China). The sample-containing tube was properly labeled and stored in an ice box maintained at a temperature lower than 8°C. All samples were immediately transported to the laboratory and subjected to microbiological analysis within 6 h. During sample collection, data were collected from staff and/or pet owners using a questionnaire that focused on assessing the possible risk factors for *Salmonella* infection.

### *Salmonella* isolation and identification

Briefly, rectal swabs in BPW pre-enrichment broth were homogenized using a vortex mixer for 30 s and incubated at 37°C for 16–24 h. A 300 µL pre-enriched suspension was added to 9.7 mL of tetrathionate broth (TTB) (HuanKai Microbial, Guangdong, China) and incubated at 37°C for 18 h. A loop of inoculum from each enrichment culture was streaked onto CHROMagar *Salmonella*-selective plate (Chromagar, Shanghai, China) at 37°C for 24–48 h. Typical mauve colonies on chromogenic plates were further confirmed by specific polymerase chain reaction (PCR) with *Taq* PCR Mix (Sangon Biotech, Shanghai, China) targeting the *invA* gene (28). The confirmed isolates were aliquoted and stored at −80°C in Luria–Bertani (LB) broth supplemented with 50% glycerol until further testing.

### *Salmonella* serotyping and MLST

Serovars of *Salmonella* isolates were confirmed with the slide agglutination test by determining the types of the O and H antigens with diagnostic sera for *Salmonella* (Tianrun, Ningbo, China), according to the Kauffmann-White scheme. The antigenic formulae of Grimont and Weill (3) were used to name the serovars.

Primers (Table S14) for the amplification of seven housekeeping genes by PCR were synthesized according to published methods (68). PCR products of amplified genes were sequenced by Sangon Biotech Co., Ltd. The sequencing results of each strain were uploaded to the MLST database (http://mlst.warwick.ac.uk/mlst/dbs/Senterica) to compare the sequence types.

### Antimicrobial susceptibility testing

The antimicrobial susceptibility of the *Salmonella* isolates was determined using the disk diffusion method on Mueller-Hinton agar plates according to the guidelines of Clinical and Laboratory Standards Institute standards (CLSI) M100-S32 (69) and VET08Ed4E (70). The following commonly used antimicrobials for pets, food-producing animals, or humans in China were assessed: ampicillin (AMP), cephalexin (LEX), cefazolin (CFZ), cefoxitin (FOX), cefotaxime (CTX), ceftriaxone (CRO), ceftazidime (CAZ), cefepime (FEP), imipenem (IPM), aztreonam (ATM), streptomycin (STR), kanamycin (KAN), gentamicin (GEN), amikacin (AMK), tetracycline (TET), doxycycline (DOX), chloramphenicol (CHL), florfenicol (FFC), nalidixic acid (NAL), norfloxacin (NOR), ciprofloxacin (CIP), enrofloxacin (ENO), ofloxacin (OFX), enoxacin (ENX), gatifloxacin (GAT), trimethoprim (TMP), sulfisoxazole (FIS), and trimethoprim-sulfamethoxazole (SXT). The diameter of the inhibition zone was measured to the nearest millimeter using a vernier caliper. The interpretation of the categories of susceptible, intermediate, or resistant was based on CLSI guidelines (69, 70). The reference strain *E. coli* ATCC 25922 was used as a quality control.

### Detection of β-lactamase genes, PMQR genes, tetracycline and sulfonamide resistance genes, and mutations within the QRDRs

PCR was used to detect the presence of β-lactamase genes, PMQR genes, tetracycline and sulfonamide resistance genes, and mutations within the QRDRs to analyze the resistance mechanisms. The primer sequences and corresponding PCR conditions are presented in Table S14. The amplified β-lactamase genes were *bla*_TEM_ (71), *bla*_CTX-M_ (72), *bla*_SHV_ (71), *bla*_OXA_ (73), *bla*_CMY_ (71), *bla*_PSE_ (74), *bla*_PER_ (74), *bla*_VEB_ (75), and *bla*_GES_ (75), whereas the amplified PMQR genes included *qnrA* (76), *qnrB* (77), *qnrC* (28), *qnrD* (28), *qnrVC* (28), *qnrS* (28), *aac(6′)-Ib-cr* (28), *oqxA* (28), *oqxB*, and *qepA* (78). Moreover, tetracycline resistance genes were identified by PCR according to reports from Ng et al. (79) (*tet*(A), *tet*(B), *tet*(C), *tet*(D), *tet*(E), *tet*(G), *tet*(M), *tet*(S), and *tet*(X)), Fan et al. (80) (*tet*(J)), and Ji et al. (81) (*tet*(X1), *tet*(X2), *tet*(X3), *tet*(X4), and *tet*(X5)). The isolates were also screened for the presence of the sulfonamide resistance genes *sul1* (71), *sul2* (71), and *sul3* (41). The QRDR genes *gyrA* and *parC* were amplified as described previously (82). PCR products were analyzed by electrophoresis on a 1.5% agarose gel, and the positive amplicons were sequenced. The sequencing results were aligned and analyzed using BLAST (http://www.ncbi.nlm.nih.gov/BLAST/). The resulting DNA sequences of all PCR products from the amplification of *gyrA* and *parC* were compared with the *Salmonella* Typhimurium LT2 (accession number: AE006468) genome sequence as a reference.

### Conjugation experiments

Conjugation experiments were conducted for isolates harboring four or more types of resistance genes by a liquid mating-out assay in LB medium using sodium azide-resistant *E. coli* J53 Az^R^ as the recipient. The donor bacterium and recipient were co-inoculated in 10 mL of LB media at 37°C for 24–48 h to facilitate plasmid conjugation transferring from the donor to the recipient. Cultured mixtures were inoculated on MacConkey plates supplemented with 100 µg/mL sodium azide, 100 µg/mL tetracycline, or 100 µg/mL ampicillin and incubated at 37°C for another 24–48 h. The presence of resistance genes was detected from the pink colonies as the suspicious transconjugants on sodium azide and tetracycline/ampicillin-containing MacConkey plates by PCR as described.

### Statistical analysis

The figure was created with Microsoft Excel version 2013. Statistical analysis was performed using GraphPad Prism (version 8.0). Differences between proportions were calculated using the chi-square test. *<*i>*P* values ≤ 0.05, 0.01, or 0.001 were considered to indicate statistical significance.

## Data Availability

The data reported in this paper have been deposited in the GenBase in National Genomics Data Center (83), Beijing Institute of Genomics, Chinese Academy of Sciences/China National Center for Bioinformation, under accession numbers C_AA062945.1–C_AA065941.1. The accession number of each gene can be checked in Table S15.

## SUPPLEMENTAL MATERIAL

Table S1 spectrum.03542-23-s0001.xlsx Fecal sample information.

Supplemental tables spectrum.03542-23-s0002.docx Tables S2-S14.

Table S15 spectrum.03542-23-s0003.xlsx Accession numbers of gene sequences.

ASM does not own the copyrights to Supplemental Material that may be linked to, or accessed through, an article. The authors have granted ASM a non-exclusive, world-wide license to publish the Supplemental Material files. Please contact the corresponding author directly for reuse.
