# Supplementary material for: Serovar and sequence type distribution and phenotypic and genotypic antimicrobial resistance of Salmonella originating from pet animals in Chongqing, China
Source: Microbiol Spectr. 2024 May 17;12(7):e03542-23. doi: 10.1128/spectrum.03542-23 (PMC11218468; doi:10.1128/spectrum.03542-23)
Supplement: Supplemental tables — Tables S2-S14. [file spectrum.03542-23-s0002.docx]

**TABLE S2** Prevalence of *Salmonella* isolated from different pet animals

| Animal species | No. of rectal samples | No. of isolates | Isolation rate |
| --- | --- | --- | --- |
| Dog | 3638 | 159 | 4.4%^a^ |
| Cat | 2409 | 90 | 3.7%^a^ |
| Turtle | 87 | 71 | 81.6%^b^ |
| Parrot | 40 | 0 |  |
| Lizard | 21 | 14 | 66.7%^c^ |
| Hedgehog | 12 | 0 |  |
| Pet rabbit | 7 | 0 |  |
| Pet rat | 6 | 0 |  |
| Fox | 2 | 0 |  |
| Snake | 1 | 0 |  |
| Total | 6223 | 334 | 5.4% |

Note: ^a,b,c^ Values in the same line with different superscript letter indicate significant differences (*p* < 0.05). Statistical significance was determined using the chi-square test.

**TABLE S3** Distribution of **s**erovars and sequence types of *Salmonella* isolates recovered from different pet animals

| **Serovar** | **Sequence type** | **Dogs (%)**  **(n=159)** | **Cats (%)**  **(n=90)** | **Turtles (%)**  **(n=71)** | **Lizards (%)**  **(n=21)** | **Total (%)**  **(n=334)** |
| --- | --- | --- | --- | --- | --- | --- |
| Typhimurium monophasic variant | ST19 | 12 (7.5) | 7 (7.8) |  |  | 39 (11.7) |
|  | ST20 | 2 (1.3) | 1 (1.1) |  |  |  |
|  | ST34 | 10 (6.3) | 6 (6.7) |  |  |  |
|  | ST469 | 1 (0.6) |  |  |  |  |
| Kentucky | ST198 | 21 (13.2) | 12 (13.3) |  |  | 34 (10.2) |
|  | ST314 | 1 (0.6) |  |  |  |  |
| Enteritidis | ST11 | 18 (11.3) | 11 (12.2) |  |  | 29 (8.7) |
| Pomona | ST451 | 1 (0.6) | 4 (4.4) | 18 (25.4) |  | 23 (6.9) |
| Give | ST155 | 13 (8.2) | 7 (7.8) |  |  | 20 (6.0) |
| Saintpaul | ST27 | 2 (1.3) |  |  |  | 15 (4.5) |
|  | ST50 | 4 (2.5) | 5 (5.6) | 4 (5.6) |  |  |
| Mbandaka | ST413 | 13 (8.2) | 1 (1.1) |  |  | 14 (4.2) |
| Typhimurium | ST19 | 5 (3.1) | 2 (2.2) |  |  | 14 (4.2) |
|  | ST34 | 4 (2.5) | 1 (1.1) |  |  |  |
|  | ST314 | 1 (0.6) |  |  |  |  |
|  | ST469 |  | 1 (1.1) |  |  |  |
| Cotham | ST617 |  | 1 (1.1) | 3 (4.2) | 7 (50.0) | 13 (3.9) |
|  | ST2259 |  |  |  | 2 (14.2) |  |
| Bovismorbificans | ST358 | 7 (7.7) | 1 (1.1) | 1 (1.4) |  | 11 (3.3) |
|  | ST1499 |  | 1 (1.1) |  |  |  |
|  | ST2259 | 1 (0.6) |  |  |  |  |
| Rissen | ST413 | 1 (0.6) |  |  |  | 11 (3.3) |
|  | ST469 | 7 (4.4) | 3 (3.3) |  |  |  |
| Takoradi | ST198 | 4 (2.5) | 7 (7.8) |  |  | 11 (3.3) |
| Derby | ST40 | 6 (3.8) | 4 (4.4) |  |  | 10 (3.0) |
| 61:z:z44 | ST287 |  |  | 3 (4.2) |  | 9 (2.7) |
|  | ST1306 | 4 (2.5) |  | 1 (1.4) |  |  |
|  | ST3038 |  |  | 1 (1.4) |  |  |
| Stanley | ST29 | 5 (3.1) | 1 (1.1) | 1 (1.4) |  | 8 (2.4) |
|  | ST2820 |  |  | 1 (1.4) |  |  |
| Kottbus | ST45 |  |  | 6 (8.5) |  | 7 (2.1) |
|  | ST808 |  |  |  | 1 (7.1) |  |
| 23:z:- | ST2889 |  |  | 6 (8.5) |  | 6 (1.8) |
| Abony | ST17 | 2 (1.3) | 3 (3.3) |  |  | 6 (1.8) |
|  | ST226 |  |  | 1 (1.4) |  |  |
| Bokanjac | ST434 |  |  | 4 (5.6) |  | 6 (1.8) |
|  | ST451 |  |  | 1 (1.4) |  |  |
|  | ST4007 |  |  |  | 1 (7.1) |  |
| Chester | ST49 | 1 (0.6) |  | 3 (4.2) |  | 5 (1.5) |
|  | ST343 | 1 (0.6) |  |  |  |  |
| Essen | ST11 | 1 (0.6) | 4 (4.4) |  |  | 5 (1.5) |
| Manhattan | ST18 |  |  | 1 (1.4) |  | 5 (1.5) |
|  | ST83 |  |  | 3 (4.2) |  |  |
|  | ST2820 |  |  | 1 (1.4) |  |  |
| Cerro | ST1593 | 1 (0.6) | 3 (3.3) |  |  | 4 (1.2) |
| 17:a:z35 | ST3918 |  |  | 3 (4.2) |  | 3 (0.9) |
| 18:z35:- | ST2924 | 1 (0.6) |  | 2 (2.8) |  | 3 (0.9) |
| 23:-:6 | ST2889 |  |  | 2 (2.8) |  | 3 (0.9) |
|  | ST5494 | 1 (0.6) |  |  |  |  |
| Newport | ST45 |  |  | 1 (1.4) |  | 3 (0.9) |
|  | ST166 |  |  | 2 (2.8) |  |  |
| Agona | ST13 |  | 2 (2.2) |  |  | 2 (0.6) |
| Ⅱ Lethe | ST440 |  |  | 1 (1.4) |  | 2 (0.6) |
|  | ST7352 | 1 (0.6) |  |  |  |  |
| Muenster | ST321 |  | 1 (1.1) |  |  | 2 (0.6) |
|  | ST684 | 1 (0.6) |  |  |  |  |
| 45:a:z35 | ST2259 |  |  |  | 1 (7.1) | 1 (0.3) |
| 50:-:- | ST2197 |  |  |  | 1 (7.1) | 1 (0.3) |
| 52:-:1,5,7 | ST1300 |  |  |  | 1 (7.1) | 1 (0.3) |
| 60:r:- | ST1197 | 1 (0.6) |  |  |  | 1 (0.3) |
| Infantis | ST32 | 1 (0.6) |  |  |  | 1 (0.3) |
| Litchfield | ST214 |  |  | 1 (1.4) |  | 1 (0.3) |
| Newlands | ST64 | 1 (0.6) |  |  |  | 1 (0.3) |
| Newrochelle | ST463 | 1 (0.6) |  |  |  | 1 (0.3) |
| Paratyphi B | ST17 | 1 (0.6) |  |  |  | 1 (0.3) |
| Senftenberg | ST14 | 1 (0.6) |  |  |  | 1 (0.3) |
| Unidentified | ST11 | 1 (0.6) |  |  |  | 1 (0.3) |

**TABLE S4** Prevalence of β-lactamase genotypes in *Salmonella* isolates originating from different pets

| **Genotype combination of β-lactamase genes** | | | **Dogs (%)**  **(n=159)** | **Cats (%)**  **(n=90)** | **Turtles (%)**  **(n=71)** | **Lizards (%)**  **(n=21)** | **Total (%)**  **(n=334)** |
| --- | --- | --- | --- | --- | --- | --- | --- |
| TEM-1 |  |  | 44 (27.7) | 25 (27.8) | 4 (5.6) |  | 73 (21.9) |
| TEM-1 | CTX-M-14 |  | 2 (1.3) |  |  |  | 2 (0.6) |
| TEM-1 | CTX-M-55 |  | 17 (10.7) | 16 (17.8) | 1 (1.4) |  | 34 (10.2) |
| TEM-1 | CTX-M-65 |  | 1 (0.6) |  |  |  | 1 (0.3) |
| TEM-1 | CTX-M-14 | OXA-10 | 2 (1.3) | 1 (1.1) |  |  | 3 (0.9) |
| TEM-116 |  |  | 14 (8.8) | 5 (5.6) | 19 (26.8) | 1 (7.1) | 39 (11.7) |
| TEM-116 | CTX-M-14 |  | 1 (0.6) |  |  |  | 1 (0.3) |
| TEM-116 |  | OXA-10 |  |  | 1 (1.4) |  | 1 (0.3) |
| TEM-171 |  | OXA-10 | 1 (0.6) |  |  |  | 1 (0.3) |
| TEM-201 |  |  | 12 (7.5) | 5 (5.6) |  |  | 17 (5.1) |
| TEM-201 | CTX-M-14 | OXA-10 | 1 (0.6) |  |  |  | 1 (0.3) |
| TEM-201 | CTX-M-55 |  | 5 (3.1) | 5 (5.6) |  |  | 10 (3.0) |
| TEM-201 | CTX-M-65 |  |  | 1 (1.1) |  |  | 1 (0.3) |
| TEM-244 |  |  | 1 (0.6) | 1 (1.1) |  |  | 2 (0.6) |
|  | CTX-M-14 |  | 2 (1.3) | 2 (2.2) |  |  | 4 (1.2) |
|  | CTX-M-14 | OXA-10 | 1 (0.6) |  |  |  | 1 (0.3) |
|  | CTX-M-14b |  | 3 (1.9) |  |  |  | 3 (0.9) |
|  | CTX-M-55 |  | 1 (0.6) | 1 (1.1) |  |  | 2 (0.6) |
|  | CTX-M-55 | OXA-10 |  | 3 (3.3) |  |  | 3 (0.9) |
|  | CTX-M-65 |  | 1 (0.6) |  |  |  | 1 (0.3) |
|  |  |  | 50 (31.4) | 25 (27.8) | 46 (64.8) | 13 (92.9) | 134 (40.1) |

**TABLE S5** Prevalence of PMQR genotype forms in *Salmonella* isolates originating from different pets

| **Genotype combination of PMQR genes** | **Dogs (%)** | **Cats (%)** | **Turtles (%)** | **Total (%)** |
| --- | --- | --- | --- | --- |
| *qnrA1*, *aac(6')-Ib-cr* | 4 (6.8) | 6 (17.6) | 5 (23.8) | 15 (13.2) |
| *qnrA1*, *qnrB6*, *aac(6')-Ib-cr* |  |  | 1 (4.8) | 1 (0.9) |
| *qnrB4*, *aac(6')-Ib-cr* |  |  | 1 (4.8) | 1 (0.9) |
| *qnrB6*, *aac(6')-Ib-cr* | 4 (6.8) |  | 6 (28.6) | 10 (8.8) |
| *qnrB6*, *qnrS2*, *aac(6')-Ib-cr* | 1 (1.7) |  |  | 1 (0.9) |
| *qnrB6*, *qnrS2*, *aac(6')-Ib-cr*, *oqxB10* |  | 1 (2.9) |  | 1 (0.9) |
| *qnrD1* | 1 (1.7) | 1 (2.9) |  | 2 (1.8) |
| *qnrD1*, *aac(6')-Ib-cr* |  |  | 1 (4.8) | 1 (0.9) |
| *qnrS2*, *aac(6')-Ib-cr* | 1 (1.7) | 2 (5.9) |  | 3 (2.6) |
| *qnrS2*, *aac(6')-Ib-cr*, *oqxB10* | 1 (1.7) |  |  | 1 (0.9) |
| *qnrS10* | 31 (52.5) | 20 (58.8) | 5 (2.4) | 56 (49.1) |
| *qnrS10*, *oqxB10* | 1 (1.7) |  |  | 1 (0.9) |
| *oqxB10*, *aac(6')-Ib-cr* | 2 (3.4) |  |  | 2 (1.8) |
| *aac(6')-Ib-cr* | 13 (22.0) | 4 (11.8) | 2 (9.5) | 19 (16.7) |
| Total | 59 | 34 | 21 | 114 |

**TABLE S6** Statistical analysis of the tetracycline resistance phenotype and genotype in *Salmonella* isolates recovered from different pets

| **Phenotype and genotype of tetracycline resistance** | **Dogs (%)** | **Cats (%)** | **Turtles (%)** | **Lizards (%)** | **Total (%)** |
| --- | --- | --- | --- | --- | --- |
| phenotype-positive/genotype-positive | 92 (57.9) | 58 (64.4) | 16 (22.5) | 1 (7.1) | 167 (50.0^a^) |
| phenotype-positive/genotype-negative | 7 (4.4) | 4 (4.4) | 3 (4.2) |  | 14 (4.2^b^) |
| phenotype-negative/genotype-positive | 5 (3.1) | 2 (2.2) | 2 ((2.8) |  | 9 (2.7^b^) |
| phenotype-negative/genotype-negative | 55 (34.6) | 26 (28.9) | 50 (70.4) | 13 (92.9) | 144 (43.1^a^) |
| Total | 159 | 90 | 71 | 14 | 334 |

Note: ^a,b^ Values in the same line with different superscript letter indicate highly significant differences (*p* < 0.01). Statistical significance was determined using the chi-square test.

**TABLE S7** Prevalence of tetracycline resistance genotypes in *Salmonella* isolates originating from different pets

| **Genotype combination of tetracycline resistance genes** | | | | | | **Dogs (%)** | **Cats (%)** | **Turtles (%)** | **Lizards (%)** | **Total (%)** |
| --- | --- | --- | --- | --- | --- | --- | --- | --- | --- | --- |
| *tet*(A) |  |  |  |  |  | 49 (30.8) | 39 (43.3) | 10 (14.1) |  | 98 (29.3) |
| *tet*(A) | *tet*(B) |  |  |  |  | 2 (1.3) | 1 (1.1) | 1 (1.4) |  | 4 (1.2) |
| *tet*(A) | *tet*(B) |  |  |  | *tet*(M) | 1 (0.6) |  |  |  | 1 (0.3) |
| *tet*(A) |  | *tet*(C) |  | *tet*(J) |  | 4 (2.5) |  |  |  | 4 (1.2) |
| *tet*(A) |  |  |  | *tet*(J) |  |  | 2 (2.2) |  |  | 2 (0.6) |
| *tet*(A) |  |  |  |  | *tet*(M) | 17 (10.7) | 6 (6.7) |  |  | 23 (6.9) |
|  | *tet*(B) |  |  |  |  | 10 (6.3) | 6 (6.7) | 2 (2.8) | 1 (7.1) | 19 (5.7) |
|  | *tet*(B) |  | *tet*(D) |  |  | 1 (0.6) |  |  |  | 1 (0.3) |
|  | *tet*(B) |  |  | *tet*(J) |  | 1 (0.6) | 1 (1.1) |  |  | 2 (0.6) |
|  |  | *tet*(C) |  | *tet*(J) |  | 2 (1.3) |  |  |  | 2 (0.6) |
|  |  |  | *tet*(D) |  |  | 7 (4.4) | 5 (5.6) | 5 (7.0) |  | 17 (5.1) |
|  |  |  |  | *tet*(J) |  | 1 (0.6) |  |  |  | 1 (0.3) |
|  |  |  |  |  | *tet*(M) | 2 (1.3) |  |  |  | 2 (0.6) |
|  |  |  |  |  |  | 62 (39.0) | 30 (33.3) | 53 (74.6) | 13 (92.9) | 158 (47.3) |
| Total |  |  |  |  |  | 159 | 90 | 71 | 14 | 334 |

**TABLE S8** Statistical analysis of the sulfonamide resistance phenotype and genotype in *Salmonella* isolates recovered from different pets

| **Phenotype and genotype of sulfonamide resistance** | **Dogs (%)** | **Cats (%)** | **Turtles (%)** | **Lizards (%)** | **Total (%)** |
| --- | --- | --- | --- | --- | --- |
| phenotype-positive/genotype-positive | 138 (86.8) | 79 (87.8) | 48 (67.6) | 12 (85.7) | 277 (82.9) |
| phenotype-positive/genotype-negative | 11 (6.9) | 4 (4.4) | 13 (18.3) | 1 (7.1) | 29 (8.7) |
| phenotype-negative/genotype-positive | 9 (5.7) | 7 (7.8) | 9 (12.7) | 1 (7.1) | 26 (7.8) |
| phenotype-negative/genotype-negative | 1 (0.6) |  | 1 (1.4) |  | 2 (0.6) |
| Total | 159 | 90 | 71 | 14 | 334 |

**TABLE S9** Prevalence of sulfonamide resistance genotypes in *Salmonella* isolates originating from different pets

| **Genotype combination of sulfonamide resistance genes** | | | **Dogs (%)** | **Cats (%)** | **Turtles (%)** | **Lizards (%)** | **Total (%)** |
| --- | --- | --- | --- | --- | --- | --- | --- |
| *sul1* |  |  | 78 (49.1) | 53 (58.9) | 54 (76.1) | 12 (85.7) | 197 (59.0) |
| *sul1* | *sul2* |  | 50 (31.4) | 24 (26.7) | 2 (2.8) | 1 (7.1) | 77 (23.1) |
| *sul1* | *sul2* | *sul3* | 5 (3.1) | 2 (2.2) | 1 (1.4) |  | 8 (2.4) |
|  | *sul2* |  | 10 (6.3) | 4 (4.4) |  |  | 14 (4.2) |
|  | *sul2* | *sul3* | 3 (1.9) | 2 (2.2) |  |  | 5 (1.5) |
|  |  | *sul3* | 1 (0.6) |  |  |  | 1 (0.3) |
|  |  |  | 12 (7.5) | 5 (5.6) | 14 (19.7) | 1 (7.1) | 32 (9.6) |
| Total |  |  | 159 | 90 | 71 | 14 | 334 |

**TABLE S10** Distribution of serovars of *Salmonella* isolates simultaneously harboring four types of resistance genes

| **Serovar** | **Dogs (%)** | **Cats (%)** | **Turtles (%)** | **Total (%)** |
| --- | --- | --- | --- | --- |
| Abony | 1/2 (50.0) | 3/3 (100.0) |  | 4/6 (66.7) |
| Bokanjac |  |  | 1/1 (100.0) | 1/6 (16.7) |
| Bovismorbificans | 6/8 (75.0) | 1/2 (50.0) |  | 7/11 (63.6) |
| Derby | 3/6 (50.0) | 3/4 (75.0) |  | 6/10 (60.0) |
| Enteritidis | 1/18 (5.6) | 1/11 (9.1) |  | 2/29 (6.9) |
| Give | 5/13 (38.5) | 4/7 (57.1) |  | 9/20 (45.0) |
| Kentucky | 7/22 (31.8) | 4/12 (33.3) |  | 11/34 (32.4) |
| Kottbus |  |  | 1/1 (100.0) | 1/7 (14.3) |
| Manhattan |  |  | 1/5 (20.0) | 1/5 (20.0) |
| Muenster |  | 1/1 (100.0) |  | 1/2 (50.0) |
| Rissen | 2/8 (25.0) | 2/3 (66.7) |  | 4/11 (36.4) |
| Saintpaul | 6/6 (100.0) | 5/5 (100.0) | 4/4 (100.0) | 15/15 (100.0) |
| Stanley |  |  | 1/2 (50.0) | 1/8 (12.5) |
| Takoradi |  | 1/7 (14.3) |  | 1/11 (9.1) |
| Typhimurium | 3/10 (30.0) | 2/4 (50.0) |  | 5/14 (35.7) |
| Typhimurium monophasic variant | 14/25 (56.0) | 3/14 (21.4) |  | 17/39 (43.6) |
| Total | 48/159 (30.2) | 30/90 (33.3) | 8/71 (11.3) | 86/334 (25.7) |

**TABLE S11** Distribution of sequence types of *Salmonella* isolates simultaneously harboring four types of resistance genes

| **Sequence type** | **Dogs (%)** | **Cats (%)** | **Turtles (%)** | **Total (%)** |
| --- | --- | --- | --- | --- |
| ST11 | 1/19 (5.3) | 1/16 (6.25) |  | 2/35 (5.7) |
| ST17 | 1/3 (33.3) | 3/3 (100.0) |  | 4/6 (66.7) |
| ST18 |  |  | 1/1 (100.0) | 1/1 (100.0) |
| ST19 | 11/17 (64.7) | 2/9 (22.2) |  | 13/26 (50.0) |
| ST27 | 2/2 (100.0) |  |  | 2/2 (100.0) |
| ST29 |  |  | 1/1 (100.0) | 1/7 (14.3) |
| ST34 | 5/14 (35.7) | 3/7 (42.9) |  | 8/21 (38.1) |
| ST40 | 3/6 (50.0) | 3/4 (75.0) |  | 6/10 (60.0) |
| ST45 |  |  | 1/7 (14.3) | 1/7 (14.3) |
| ST50 | 4/4 (100.0) | 5/5 (100.0) | 4/4 (100.0) | 13/13 (100.0) |
| ST155 | 5/13 (38.5) | 4/7 (57.1) |  | 9/20 (45.0) |
| ST198 | 6/25 (24.0) | 5/19 (26.3) |  | 11/44 (25.0) |
| ST314 | 1/2 (50.0) |  |  | 1/2 (50.0) |
| ST321 |  | 1/1 (100.0) |  | 1/1 (100.0) |
| ST358 | 6/7 (85.7) | 1/1 (100.0) |  | 7/9 (77.8) |
| ST451 |  |  | 1/19 (5.3) | 1/24 (4.2) |
| ST469 | 3/8 (37.5) | 2/4 (50.0) |  | 5/12 (41.7) |
| Total | 48/159 (30.2) | 30/90 (33.3) | 8/71 (11.3) | 86/334 (25.7) |

**Table S12** Transfer of the resistance gene from donor bacteria harboring four resistance genes to the recipient *E. coli* J53 Az^R^

| **Strain** | **Host** | **Serovar** | **Sequence type** | **Resistance genes harbored** | **Cotransferred resistance gene** |
| --- | --- | --- | --- | --- | --- |
| CQBB2019DS001 | Dog | Give | ST155 | *bla*_TEM-1_, *aac(6')-Ib-cr*, *tet*(A), *sul1*, *sul2* | *aac(6')-Ib-cr*, *sul1*, *sul2* |
| CQBB2019DS002 | Dog | Give | ST155 | *bla*_TEM-1_, *aac(6')-Ib-cr*, *tet*(A), *sul1*, *sul2* | *aac(6')-Ib-cr, sul1, sul2* |
| CQYB2020DS002 | Dog | Give | ST155 | *bla*_TEM-1_, *qnrB6*, *aac(6')-Ib-cr*, *tet*(A), *sul1*, *sul2* | *bla*_TEM-1_, *qnrB6*, *aac(6')-Ib-cr*, *tet*(A), *sul1*, *sul2* |
| CQBB2020DS006 | Dog | Kentucky | ST314 | *bla*_TEM-116_, *aac(6')-Ib-cr*, *tet*(A), *sul1* | *bla*_TEM-116_, *aac(6')-Ib-cr*, *tet*(A), *sul1* |
| CQYZ2020DS013 | Dog | Typhimurium monophasic variant | ST19 | *bla*_TEM-116_, *bla*_CTX-M-14_, *qnrS2*, *aac(6')-Ib-cr*, *tet*(A), *sul1*, *sul2* | *bla*_TEM-116_, *bla*_CTX-M-14_, *qnrS2*, *aac(6')-Ib-cr*, *tet*(A), *sul1*, *sul2* |
| CQYB2020CS017 | Dog | Derby | ST40 | *bla*_TEM-1_, *qnrS10*, *tet*(A), *tet*(M), *sul1*, *sul2*, *sul3* | *bla*_TEM-1_, *tet*(A), *sul1* |
| CQJB2020DS032 | Dog | Saintpaul | ST50 | *bla*_TEM-1_, *qnrA1*, *aac(6')-Ib-cr*, *tet*(D), *sul1* | *aac(6')-Ib-cr, sul1* |
| CQJB2020DS033 | Dog | Saintpaul | ST50 | *bla*_TEM-171_, *bla*_OXA-10_, *qnrA1*, *aac(6')-Ib-cr*, *tet*(D), *sul1* | *aac(6')-Ib-cr, sul1* |
| CQYB2020DS038 | Dog | Saintpaul | ST50 | *bla*_TEM-201_, *qnrA1*, *aac(6')-Ib-cr*, *tet*(D), *sul1* | *qnrA1, aac(6')-Ib-cr, sul1* |
| CQYB2020DS055 | Dog | Typhimurium | ST34 | *bla*_TEM-1_, *bla*_CTX-M-14_, *bla*_OXA-10_, *qnrS10*, *tet*(A), *tet*(B), *sul1*, *sul2* | *bla*_TEM-1_, *bla*_CTX-M-14_, *bla*_OXA-10_, *qnrS10*, *tet*(A), *sul1*, *sul2* |
| CQBB2020DS058 | Dog | Bovismorbificans | ST358 | *bla*_TEM-1_, *qnrS10*, *tet*(M), *sul1*, *sul2*, *sul3* | *bla*_TEM-1_, *tet*(M), *sul1*, *sul2* |
| CQBB2020DS062 | Dog | Saintpaul | ST50 | *bla*_TEM-116_, *qnrB6*, *aac(6')-Ib-cr*, *tet*(A), *sul1* | *bla*_TEM-116_, *qnrB6*, *sul1* |
| CQBB2020DS075 | Dog | Typhimurium monophasic variant | ST19 | *bla*_TEM-201_, *bla*_CTX-M-14_, *bla*_OXA-10_, *qnrS10*, *tet*(A), *sul1* | *bla*_CTX-M-14_, *bla*_OXA-10_, *sul1* |
| CQJB2020DS080 | Dog | Rissen | ST469 | *bla*_TEM-201_, *qnrS10*, *tet*(A), *tet*(M), *sul1*, *sul2*, *sul3* | *bla*_TEM-201_, *tet*(A), *sul1*, *sul2* |
| CQBB2020DS093 | Dog | Give | ST155 | *bla*_TEM-1_, *qnrB6*, *aac(6')-Ib-cr*, *tet*(A), *sul1*, *sul2* | *aac(6')-Ib-cr, tet*(A), *sul1*, *sul2* |
| CQBB2020DS095 | Dog | Give | ST155 | *bla*_TEM-1_, *aac(6')-Ib-cr*, *tet*(A), *sul1*, *sul2* | *aac(6')-Ib-cr, tet*(A), *sul1*, *sul2* |
| CQYB2020DS097 | Dog | Typhimurium monophasic variant | ST19 | *bla*_TEM-1_, *qnrS10*, *tet*(A), *tet*(B), *tet*(M), *sul2*, *sul3* | *bla*_TEM-1_, *tet*(B) |
| CQBB2021DS027 | Dog | Typhimurium monophasic variant | ST19 | *bla*_TEM-201_, *qnrS10*, *tet*(A), *tet*(M), *sul1*, *sul2* | *bla*_TEM-201_, *tet*(A), *sul1*, *sul2* |
| CQBB2021DS028 | Dog | Typhimurium monophasic variant | ST19 | *bla*_TEM-201_, *qnrS10*, *tet*(A), *tet*(M), *sul1*, *sul2* | *bla*_TEM-201_, *tet*(A), *tet*(M), *sul1*, *sul2* |
| CQBB2021DS029 | Dog | Typhimurium monophasic variant | ST19 | *bla*_TEM-201_, *qnrS10*, *tet*(A), *tet*(M), *sul2* | *tet*(A), *sul2* |
| CQBB2021DS041 | Dog | Saintpaul | ST27 | *bla*_TEM-201_, *bla*_CTX-M-55_, *qnrS10*, *tet*(A), *sul1* | *bla*_CTX-M-55_, *tet*(A), *sul1* |
| CQBB2021DS042 | Dog | Saintpaul | ST27 | *bla*_TEM-201_, *bla*_CTX-M-55_, *qnrS10*, *tet*(A), *sul1* | *bla*_TEM-201_, *bla*_CTX-M-55_, *qnrS10*, *tet*(A), *sul1* |
| CQBB2021DS043 | Dog | Typhimurium monophasic variant | ST19 | *bla*_TEM-201_, *qnrS10*, *tet*(A), *tet*(M), *sul2*, | *bla*_TEM-201_, *tet*(A) |
| CQBB2021DS044 | Dog | Typhimurium monophasic variant | ST19 | *bla*_TEM-201_, *qnrS10*, *tet*(A), *tet*(M), *sul1*, *sul2* | *sul1, sul2* |
| CQYB2021DS050 | Dog | Typhimurium monophasic variant | ST19 | *bla*_TEM-201_, *qnrS10*, *tet*(A), *tet*(M), *sul2* | *bla*_TEM-201_, *tet*(A) |
| CQBB2021DS052 | Dog | Rissen | ST469 | *bla*_TEM-1_, *bla*_CTX-M-14_, *aac(6')-Ib-cr*, *tet*(A), *tet*(C), *tet*(J), *sul1*, *sul2*, *sul3* | *bla*_TEM-1_, *bla*_CTX-M-14_, *aac(6')-Ib-cr*, *tet*(C), *tet*(J), *sul1* |
| CQJB2021DS054 | Dog | Typhimurium | ST34 | *bla*_TEM-1_, *aac(6')-Ib-cr*, *tet*(B), *tet*(D), *sul1*, *sul2* | *sul1, sul2* |
| CQYB2021DS058 | Dog | Abony | ST17 | *bla*_TEM-1_, *bla*_CTX-M-55_, *aac(6')-Ib-cr*, *oqxB10*, *tet*(A), *sul1* | *aac(6')-Ib-cr, oqxB10, tet(A), sul1* |
| CQYB2021DS073 | Dog | Typhimurium monophasic variant | ST34 | *bla*_TEM-1_, *bla*_CTX-M-55_, *qnrS10*, *tet*(B), *sul2*, *sul3* | *bla*_CTX-M-55_, *sul2* |
| CQJLP2021DS078 | Dog | Typhimurium monophasic variant | ST34 | *bla*_TEM-1_, *qnrS10*, *tet*(B), *sul2*, *sul3* | *tet*(B), *sul2* |
| CQYB2021DS099 | Dog | Kentucky | ST198 | *bla*_TEM-1_, *bla*_CTX-M-65_, *aac(6')-Ib-cr*, *tet*(A), *tet*(C), *tet*(J) *sul1*, *sul2* | *bla*_TEM-1_, *bla*_CTX-M-65_, *tet*(A), *sul1*, *sul2* |
| CQJB2021DS114 | Dog | Kentucky | ST198 | *bla*_TEM-1_, *bla*_CTX-M-55_, *qnrS10*, *tet*(A), *sul1* | *bla*_TEM-1_, *bla*_CTX-M-55_, *tet*(A), *sul1* |
| CQYB2021DS136 | Dog | Kentucky | ST198 | *bla*_CTX-M-14b_, *aac(6')-Ib-cr*, *tet*(A), *sul1* | *aac(6')-Ib-cr, tet*(A)*, sul1* |
| CQBB2021DS141 | Dog | Typhimurium monophasic variant | ST469 | *bla*_TEM-1_, *qnrS10*, *tet*(A), *tet*(M), *sul1* | *bla*_TEM-1_, *tet*(A), *sul1* |
| CQYB2021DS161 | Dog | Kentucky | ST198 | *bla*_TEM-1_, *bla*_CTX-M-55_, *qnrS10*, *tet*(A), *sul1* | *bla*_CTX-M-55_, *qnrS10*, *tet*(A), *sul1* |
| CQYB2021DS162 | Dog | Kentucky | ST198 | *bla*_TEM-1_, *bla*_CTX-M-55_, *qnrS10*, *tet*(A), *sul1* | *bla*_TEM-1_, *bla*_CTX-M-55_, *tet*(A), *sul1* |
| CQYB2021DS163 | Dog | Typhimurium monophasic variant | ST34 | *bla*_TEM-1_, *bla*_CTX-M-14_, *bla*_OXA-10_, *qnrS10*, *tet*(A), *tet*(B), *sul1*, *sul2* | *bla*_CTX-M-14_, *bla*_OXA-10_, *sul1*, *sul2* |
| CQYB2021DS167 | Dog | Derby | ST40 | *bla*_TEM-1_, *qnrB6*, *qnrS2*, *aac(6')-Ib-cr*, *tet*(A), *sul1*, *sul2* | *aac(6')-Ib-cr, tet*(A)*, sul1, sul2* |
| CQYB2021DS168 | Dog | Kentucky | ST198 | *bla*_TEM-1_, *qnrB6*, *aac(6')-Ib-cr*, *tet*(A), *sul1*, *sul2* | *qnrB6, aac(6')-Ib-cr, sul1, sul2* |
| CQBB2021DS172 | Dog | Enteritidis | ST11 | *bla*_TEM-1_, *qnrA1*, *aac(6')-Ib-cr*, *tet*(B), *tet*(J), *sul1*, *sul2* | *bla*_TEM-1_, *aac(6')-Ib-cr*, *tet*(B), *sul1*, *sul2* |
| CQBB2021DS173 | Dog | Derby | ST40 | *bla*_CTX-M-14_, *bla*_OXA-10_, *qnrS10*, *tet*(A), *tet*(M), *sul1*, *sul2* | *bla*_CTX-M-14_, *bla*_OXA-10_, *qnrS10*, *tet*(A), *sul1*, *sul2* |
| CQBB2021DS181 | Dog | Typhimurium monophasic variant | ST19 | *bla*_TEM-1_, *qnrS10*, *tet*(A), *tet*(M), *sul1*, *sul2* | *qnrS10, sul1, sul2* |
| CQJB2021DS186 | Dog | Typhimurium | ST19 | *bla*_TEM-1_, *qnrS10*, *tet*(A), *tet*(M), *sul1*, *sul2*, | *bla*_TEM-1_, *tet*(A), *sul1*, *sul2* |
| CQBB18DS001 | Dog | Bovismorbificans | ST358 | *bla*_TEM-1_, *qnrS10*, *tet*(A), *tet*(M), *sul2* | *bla*_TEM-1_, *tet*(A), *sul2* |
| CQBB18DS002 | Dog | Bovismorbificans | ST358 | *bla*_TEM-1_, *qnrS10*, *tet*(A), *tet*(M), *sul1*, *sul2* | *bla*_TEM-1_, *tet*(A), *sul1*, *sul2* |
| CQBB18DS003 | Dog | Bovismorbificans | ST358 | *bla*_TEM-1_, *qnrS10*, *tet*(A), *tet*(M), *sul1*, *sul2* | *bla*_TEM-1_, *tet*(A), *sul2* |
| CQBB18DS004 | Dog | Bovismorbificans | ST358 | *bla*_TEM-1_, *qnrS10*, *tet*(A), *tet*(M), *sul1*, *sul2* | *tet*(A), *sul1*, *sul2* |
| CQBB18DS005 | Dog | Bovismorbificans | ST358 | *bla*_TEM-1_, *qnrS10*, *tet*(A), *tet*(M), *sul1*, *sul2* | *bla*_TEM-1_, *tet*(A), *sul1*, *sul2* |
| CQBB2020CS001 | Cat | Bovismorbificans | ST358 | *bla*_TEM-1_, *qnrS10*, *tet*(A), *tet*(M), *sul1*, *sul2*, *sul3* | *qnrS10, tet*(A), *sul1*, *sul2* |
| CQYB2020CS018 | Cat | Give | ST155 | *bla*_TEM-1_, *aac(6')-Ib-cr*, *tet*(A), *sul1*, *sul2* | *bla*_TEM-1_, *aac(6')-Ib-cr*, *tet*(A), *sul1*, *sul2* |
| CQJB2020CS034 | Cat | Saintpaul | ST50 | *bla*_TEM-116_, *qnrA1*, *aac(6')-Ib-cr*, *tet*(D), *sul1* | *bla*_TEM-116_, *qnrA1*, *aac(6')-Ib-cr*, *tet*(D), *sul1* |
| CQYB2020CS035 | Cat | Saintpaul | ST50 | *bla*_TEM-1_, *qnrA1*, *aac(6')-Ib-cr*, *tet*(D), *sul1* | *bla*_TEM-1_, *qnrA1*, *aac(6')-Ib-cr*, *tet*(D), *sul1* |
| CQYB2020CS036 | Cat | Saintpaul | ST50 | *bla*_TEM-1_, *qnrA1*, *aac(6')-Ib-cr*, *tet*(D), *sul1* | *bla*_TEM-1_, *qnrA1*, *aac(6')-Ib-cr*, *tet*(D), *sul1* |
| CQYB2020CS037 | Cat | Saintpaul | ST50 | *bla*_TEM-1_, *qnrA1*, *aac(6')-Ib-cr*, *tet*(D), *sul1* | *bla*_TEM-1_, *qnrA1*, *aac(6')-Ib-cr*, *tet*(D) |
| CQYB2020CS039 | Cat | Saintpaul | ST50 | *bla*_TEM-1_, *qnrA1*, *aac(6')-Ib-cr*, *tet*(D), *sul1* | *bla*_TEM-1_, *qnrA1*, *aac(6')-Ib-cr*, *tet*(D), *sul1* |
| CQBB2020CS041 | Cat | Enteritidis | ST11 | *bla*_TEM-1_, *bla*_CTX-M-55_, *qnrA1*, *aac(6')-Ib-cr*, *tet*(B), *tet*(J) *sul1*, *sul2* | *bla*_TEM-1_, *bla*_CTX-M-55_, *aac(6')-Ib-cr*, *tet(*J), *sul1*, *sul2* |
| CQYB2020CS049 | Cat | Kentucky | ST198 | *bla*_TEM-1_, *bla*_CTX-M-55_, *qnrS10*, *tet*(A), *sul1* | *bla*_TEM-1_, *bla*_CTX-M-55_, *qnrS10*, *tet*(A), *sul1* |
| CQYB2020CS050 | Cat | Kentucky | ST198 | *bla*_TEM-1_, *bla*_CTX-M-55_, *qnrS10*, *tet*(A), *sul1* | *bla*_TEM-1_, *bla*_CTX-M-55_, *tet*(A), *sul1* |
| CQYZ2020CS052 | Cat | Typhimurium monophasic variant | ST19 | *bla*_TEM-1_, *qnrS10*, *tet*(A), *sul2*, *sul3* | *tet*(A), *sul2* |
| CQYZ2020CS054 | Cat | Typhimurium | ST19 | *bla*_TEM-201_, *qnrS10*, *tet*(A), *sul2*, *sul3* | *bla*_TEM-201_, *tet*(A), *sul2* |
| CQYZ2020CS064 | Cat | Typhimurium monophasic variant | ST34 | *bla*_TEM-1_, *bla*_CTX-M-14_, *bla*_OXA-10_, *qnrS10*, *tet*(A), *tet*(B), *sul2* | *bla*_TEM-1_, *bla*_OXA-10_, *tet*(A), *tet*(B), *sul2* |
| CQYZ2020CS067 | Cat | Give | ST155 | *bla*_TEM-1_, *qnrS10*, *tet*(A), *sul1* | *bla*_TEM-1_, *qnrS10*, *tet*(A), *sul1* |
| CQBB2020CS071 | Cat | Kentucky | ST198 | *bla*_TEM-1_, *bla*_CTX-M-55_, *qnrS10*, *tet*(A), *sul1* | *bla*_TEM-1_, *bla*_CTX-M-55_, *qnrS10*, *tet*(A), *sul1* |
| CQBB2020CS094 | Cat | Rissen | ST469 | *bla*_TEM-244_, *qnrS10*, *tet*(A), *sul1* | *bla*_TEM-244_, *tet*(A) |
| CQSPB2021CS008 | Cat | Typhimurium monophasic variant | ST34 | *bla*_TEM-201_, *bla*_CTX-M-65_, *qnrS2*, *aac(6')-Ib-cr*, *tet*(B), *sul1*, *sul2*, *sul3* | *bla*_TEM-201_, *bla*_CTX-M-65_, *aac(6')-Ib-cr*, *tet*(B), *sul1*, *sul2* |
| CQYB2021CS033 | Cat | Rissen | ST469 | *bla*_TEM-201_, *qnrS10*, *tet*(A), *tet*(M), *sul1*, *sul2* | *bla*_TEM-201_, *qnrS10*, *tet*(A), *tet*(M), *sul1*, *sul2* |
| CQYB2021CS047 | Cat | Give | ST155 | *bla*_TEM-1_, *aac(6')-Ib-cr*, *tet*(A), *tet*(J), *sul1*, *sul2* | *bla*_TEM-1_, *aac(6')-Ib-cr*, *tet*(A), *tet*(J), *sul1*, *sul2* |
| CQYB2021CS094 | Cat | Muenster | ST321 | *bla*_TEM-1_, *bla*_CTX-M-55_, *qnrS10*, *tet*(A), *sul1* | *bla*_TEM-1_, *bla*_CTX-M-55_, *qnrS10*, *tet*(A), *sul1* |
| CQYB2021CS122 | Cat | Kentucky | ST198 | *bla*_TEM-201_, *bla*_CTX-M-55_, *qnrS10*, *tet*(A), *sul1* | *bla*_TEM-201_, *bla*_CTX-M-55_, *qnrS10*, *tet*(A), *sul1* |
| CQYB2021CS160 | Cat | Takoradi | ST198 | *bla*_CTX-M-55_, *qnrS10*, *tet*(A), *sul1* | *bla*_CTX-M-55_, *qnrS10*, *tet*(A), *sul1* |
| CQYB2021CS182 | Cat | Give | ST155 | *bla*_TEM-1_, *qnrB6*, *qnrS2*, *aac(6')-Ib-cr*, *oqxB10*, *tet*(A), *sul1*, *sul2* | *bla*_TEM-1_, *qnrS2*, *aac(6')-Ib-cr*, *oqxB10*, *tet*(A), *sul1*, *sul2* |
| CQSPB2021CS187 | Cat | Abony | ST17 | *bla*_CTX-M-55_, *bla*_OXA-10_, *qnrS10*, *tet*(A), *sul1* | *bla*_CTX-M-55_, *bla*_OXA-10_, *qnrS10*, *tet*(A), *sul1* |
| CQSPB2021CS188 | Cat | Abony | ST17 | *bla*_CTX-M-55_, *bla*_OXA-10_, *qnrS10*, *tet*(A), *sul1* | *bla*_CTX-M-55_, *bla*_OXA-10_, *qnrS10*, *tet*(A), *sul1* |
| CQSPB2021CS189 | Cat | Abony | ST17 | *bla*_CTX-M-55_, *bla*_OXA-10_, *qnrS10*, *tet*(A), *sul1* | *bla*_CTX-M-55_, *bla*_OXA-10_, *qnrS10*, *tet*(A), *sul1* |
| CQBB2021CS194 | Cat | Typhimurium | ST34 | *bla*_TEM-1_, *aac(6')-Ib-cr*, *tet*(B), *sul1*, *sul2* | *bla*_TEM-1_, *tet*(B), *sul1*, *sul2* |
| CQBB9CS001 | Cat | Derby | ST40 | *bla*_TEM-1_, *qnrS10*, *tet*(A), *tet*(M), *sul1*, *sul2* | *bla*_TEM-1_, *qnrS10*, *tet*(A), *tet*(M)*, sul1*, *sul2* |
| CQBB9CS003 | Cat | Derby | ST40 | *bla*_TEM-1_, *qnrS10*, *tet*(A), *tet*(M), *sul1*, *sul2* | *bla*_TEM-1_, *qnrS10*, *tet*(A), *tet*(M), *sul1*, *sul2* |
| CQBB9CS005 | Cat | Derby | ST40 | *bla*_TEM-1_, *qnrS10*, *tet*(A), *tet*(M), *sul1*, *sul2* | *bla*_TEM-1_, *tet*(A), *sul2* |
| CQJB2020TS007 | Turtle | Stanley | ST29 | *bla*_TEM-116_, *bla*_OXA-10_, *qnrS10*, *tet*(A), *sul1* | *bla*_OXA-10_, *tet*(A), *sul1* |
| CQBB2020TS012 | Turtle | Saintpaul | ST50 | *bla*_TEM-1_, *aac(6')-Ib-cr*, *tet*(D), *sul1* | *bla*_TEM-1_, *aac(6')-Ib-cr*, *tet*(D), *sul1* |
| CQJB2020TS028 | Turtle | Saintpaul | ST50 | *bla*_TEM-1_, *qnrA1*, *aac(6')-Ib-cr*, *tet*(D), *sul1* | *bla*_TEM-1_, *qnrA1*, *aac(6')-Ib-cr*, *tet*(D), *sul1* |
| CQJB2020TS030 | Turtle | Saintpaul | ST50 | *bla*_TEM-1_, *qnrA1*, *aac(6')-Ib-cr*, *tet*(D), *sul1* | *bla*_TEM-1_, *qnrA1*, *aac(6')-Ib-cr*, *tet*(D), *sul1* |
| CQJB2020TS031 | Turtle | Saintpaul | ST50 | *bla*_TEM-116_, *qnrA1*, *aac(6')-Ib-cr*, *tet*(D), *sul1* | *bla*_TEM-116_, *qnrA1*, *aac(6')-Ib-cr*, *tet*(D), *sul1* |
| CQBB2020TS081 | Turtle | Bokanjac | ST451 | *bla*_TEM-116_, *qnrA1*, *qnrB6*, *aac(6')-Ib-cr*, *tet*(A), *tet*(B), *sul1* | *qnrA1, qnrB6, tet*(A), *sul1* |
| CQJLP2020TS104 | Turtle | Manhattan | ST18 | *bla*_TEM-1_, *qnrB6*, *aac(6')-Ib-cr*, *tet*(A), *sul1* | *bla*_TEM-1_, *qnrB6*, *aac(6')-Ib-cr*, *tet*(A), *sul1* |
| CQSPB2021TS020 | Turtle | Kottbus | ST45 | *bla*_TEM-116_, *qnrB4*, *aac(6')-Ib-cr*, *tet*(D), *sul1*, *sul2* | *bla*_TEM-116_, *qnrB4*, *aac(6')-Ib-cr*, *tet*(D), *sul1*, *sul2* |

**Table S13** Number of transferred resistance genes from 86 isolates harboring four types of resistance genes

| **Gene** | **Dogs (%)** | **Cats (%)** | **Turtles (%)** | **Total (%)** |
| --- | --- | --- | --- | --- |
| *bla*_TEM-1_ | 16/31 (51.6) | 18/20 (90.0) | 4/4 (100.0) | 38/55 (69.1) |
| *bla*_TEM-116_ | 3/3 (100.0) | 1/1 (100.0) | 2/4 (50.0) | 6/8 (75.0) |
| *bla*_TEM-201_ | 6/11 (54.5) | 4/4 (100.0) | 0 | 10/15 (66.7) |
| *bla*_TEM-244_ | 0 | 1/1(100.0) | 0 | 1/1 (100.0) |
| *bla*_CTX-M-14_ | 6/7 (85.7) | 0/1 (0) | 0 | 6/8 (75.0) |
| *bla*_CTX-M-55_ | 6/7 (85.7) | 10/10 (100.0) | 0 | 16/17 (94.1) |
| *bla*_CTX-M-65_ | 1/1 (100.0) | 1/1 (100.0) | 0 | 2/2 (100.0) |
| *bla*_OXA-10_ | 4/4 (100.0) | 4/5 (80.0) | 1/1 (100.0) | 9/10 (90.0) |
| *qnrA1* | 1/4 (25.0) | 5/6 (83.3) | 4/4 (100.0) | 10/14 (71.4) |
| *qnrB4* | 0 | 0 | 1/1 (100.0) | 1/1 (100.0) |
| *qnrB6* | 3/5 (60.0) | 0/1 (0) | 2/2 (100.0) | 5/8 (62.5) |
| *qnrS2* | 1/2 (50.0) | 1/2 (50.0) | 0 | 2/4 (50.0) |
| *qnrS10* | 5/29 (17.2) | 13/19 (68.4) | 0/1 (0) | 18/49 (36.7) |
| *oqxB10* | 1/1 (100.0) | 1/1 (100.0) | 0 | 2/2 (100.0) |
| *aac(6')-Ib-cr* | 16/19 (84.2) | 10/11 (90.9) | 6/7 (85.7) | 32/37 (86.5) |
| *tet*(A) | 30/40 (75.0) | 22/22 (100.0) | 3/3 (100.0) | 55/65 (84.6) |
| *tet*(B) | 3/7 (42.9) | 3/4 (75.0) | 0/1 (0) | 6/12 (50.0) |
| *tet*(C) | 1/2 (50.0) | 0 | 0 | 1/2 (50.0) |
| *tet*(D) | 0/4 (0) | 5/5 (100.0) | 5/5 (100.0) | 10/14 (71.4) |
| *tet*(J) | 1/3 (33.3) | 2/2 (100.0) | 0 | 3/5 (60.0) |
| *tet*(M) | 2/19 (10.5) | 3/5 (60.0) | 0 | 5/24 (20.8) |
| *sul1* | 40/41 (97.6) | 24/27 (88.9) | 8/8 (100.0) | 72/76 (97.3) |
| *sul2* | 29/34 (85.3) | 14/14 (100.0) | 1/1 (100.0) | 44/49 (89.8) |

**TABLE S14** Primer sequences and parameters used for the detection of antimicrobial resistance genes in *Salmonella* in this study

| **gene** | **Primer name** | **Primer sequence（5'-3'）** | **PCR product size (bp)** | **Annealing temperature (℃)** | **References** |
| --- | --- | --- | --- | --- | --- |
| *aroC* | aroC**-**F | CCTGGCACCTCGCGCTATAC | 826 | 60 | 73 |
|  | aroC**-**R | CCACACACGGATCGTGGCG |  |  |  |
| *dnaN* | dnaN**-**F | ATGAAATTTACCGTTGAACGTGA | 833 | 58 | 73 |
|  | dnaN**-**R | AATTTCTCATTCGAGAGGATTGC |  |  |  |
| *hemD* | hemD**-**F | GAAGCGTTAGTGAGCCGTCTGCG | 666 | 60 | 73 |
|  | hemD**-**R | ATCAGCGACCTTAATATCTTGCCA |  |  |  |
| *hisD* | hisD**-**F | GAAACGTTCCATTCCGCGCAGAC | 894 | 60 | 73 |
|  | hisD**-**R | CTGAACGGTCATCCGTTTCTG |  |  |  |
| *purE* | purE**-**F | ATGTCTTCCCGCAATAATCC | 510 | 58 | 73 |
|  | purE**-**R | TCATAGCGTCCCCCGCGGATC |  |  |  |
| *sucA* | sucA**-**F | AGCACCGAAGAGAAACGCTG | 643 | 60 | 73 |
|  | sucA**-**R | GGTTGTTGATAACGATACGTAC |  |  |  |
| *thrA* | thrA**-**F | GTCACGGTGATCGATCCGGT | 852 | 60 | 73 |
|  | thrA**-**R | CACGATATTGATATTAGCCCG |  |  |  |
| *bla*_TEM_ | TEM-F | CAGCGGTAAGATCCTTGAGA | 643 | 55 | 76 |
|  | TEM-R | ACTCCCCGTCGTGTAGATAA |  |  |  |
| *bla*_CTX-M_ | CTX-M-F | ATGTGCAGYACCAGTAARGTKATGGC | 593 | 57 | 77 |
|  | CTX-M-R | TGGGTRAARTARGTSACCAGAAYCAGCGG |  |  |  |
| *bla*_SHV_ | SHV-F | GGCCGCGTAGGCATGATAGA | 714 | 58 | 76 |
|  | SHV-R | CCCGGCGATTTGCTGATTTC |  |  |  |
| *bla*_OXA_ | OXA-1-F | ACACAATACATATCAACTTCGC | 814 | 60 | 78 |
|  | OXA-1-R | AGTGTGTGTTTAGAATGGTGATC |  |  |  |
|  | OXA-2-F | TTCAAGCCAAAGGCACGATAG | 704 | 55 | 78 |
|  | OXA-2-R | TCCGAGTTGACTGCCGGGTTG |  |  |  |
|  | OXA-10-F | CGTGCTTTGTAAAAGTAGCAG | 651 | 55 | 78 |
|  | OXA-10-R | CATGATTTTGGT GGGAATGG |  |  |  |
| *bla*_PSE_ | PSE-F | AATGGCAATCAGCGCTTCCC | 598 | 58 | 79 |
|  | PSE-R | GGGGCTTGATGCTCACTACA |  |  |  |
| *bla*_PER_ | PER-F | ATGAATGTCATCACAAAATG | 927 | 56 | 79 |
|  | PER-R | TCAATCCGGACTCACT |  |  |  |
| *bla*_VEB_ | VEB-F | CATTTCCCGATGCAAAGCGT | 648 | 55 | 80 |
|  | VEB-R | CGAAGTTTCTTTGGACTCTG |  |  |  |
| *bla*_GES_ | GES-F | AGTCGGCTAGACCGGAAAG | 399 | 55 | 80 |
|  | GES-R | TTTGTCCGTGCTCAGGAT |  |  |  |
| *qnrA* | qnrA-F | ATTTCTCACGCCAGGATTTG | 516 | 60 | 81 |
|  | qnrA-R | GATCGGCAAAGGTTAGGTCA |  |  |  |
| *qnrB* | qnrB-F | GGCATTGAAATTCGCCACTG | 263 | 61 | 82 |
|  | qnrB-R | TTTGCTGCTCGCCAGTCGAA |  |  |  |
| *qnrC* | qnrC-F | GGGTTGTACATTTATTGAATCGAATG | 447 | 55 | 28 |
|  | qnrC-R | TCCACTTTACGAGGTTCTAAGCC |  |  |  |
| *qnrD* | qnrD-F | CGAGATCAATTTACGGGGAATAG | 596 | 57 | 28 |
|  | qnrD-R | ACACCTAAACTCTCAACAAGCTGAA |  |  |  |
| *qnrVC* | qnrVC136-F | AATCAAAGCAATTATATAATCAAGTGAAC | 650 | 55 | 28 |
|  | qnrVC136-R | TTAGTCAGGAACAATGATTACCC |  |  |  |
|  | qnrVC457-F | ATGGATAAAACAGACCAGTTATATGTA | 657 | 55 | 28 |
|  | qnrVC457-R | TTAGTCAGGAACTACTATTAAACCTAAT |  |  |  |
| *qnrS* | qnrS-F | CGTCAACTGCAAGTTCATTGAAC | 435 | 57 | 28 |
|  | qnrS-R | TCTAAACCGTCGAGTTCGGC |  |  |  |
| *aac(6′)-Ib-cr* | aac(6′)-Ib-cr-F | CGCAAAAACAAAGTTAGGCATCA | 571 | 57 | 28 |
|  | aac(6′)-Ib-cr-R | CTCGAATGCCTGGCGTGTTT |  |  |  |
| *oqxA* | oqxA-F | ATGAGCCTGCAAAAAACCTGG | 1176 | 60 | 28 |
|  | oqxA-R | TCAGTTAAGGGTGGCGCTGG |  |  |  |
| *oqxB* | oqxB-F | ACCAACACGCCGAATACCG | 737 | 63 | This study |
|  | oqxB -R | ACGGATCTCAGCTTCCAGCA |  |  |  |
| *qepA* | qepA-F | GCAGGTCCAGCAGCGGGTAG | 199 | 65 | 83 |
|  | qepA-R | CTTCCTGCCCGAGTATCGTG |  |  |  |
| *gyrA* | gyrA-F | CGTTGGTGACGTAATCGGTA | 251 | 60 | 87 |
|  | gyrA-R | CCGTACCGTCATAGTTATCC |  |  |  |
| *parC* | parC-F | GACGGCCTGAAGCCGGT | 284 | 65 | 87 |
|  | parC-R | CTCGGCGTATTTGGACAGG |  |  |  |
| *tet*(A) | tet(A)-F | GCTACATCCTGCTTGCCTTC | 210 | 55 | 84 |
|  | tet(A)-R | CATAGATCGCCGTGAAGAGG |  |  |  |
| *tet*(B) | tet(B)-F | TTGGTTAGGGGCAAGTTTTG | 659 | 55 | 84 |
|  | tet(B)-R | GTAATGGGCCAATAACACCG |  |  |  |
| *tet*(C) | tet(C)-F | CTTGAGAGCCTTCAACCCAG | 418 | 55 | 84 |
|  | tet(C)-R | ATGGTCGTCATCTACCTGCC |  |  |  |
| *tet*(D) | tet(D)-F | AAACCATTACGGCATTCTGC | 787 | 55 | 84 |
|  | tet(D)-R | GACCGGATACACCATCCATC |  |  |  |
| *tet*(E) | tet(E)-F | AAACCACATCCTCCATACGC | 278 | 55 | 84 |
|  | tet(E)-R | AAATAGGCCACAACCGTCAG |  |  |  |
| *tet*(G) | tet(G)-F | GCTCGGTGGTATCTCTGCTC | 468 | 55 | 84 |
|  | tet(G)-R | AGCAACAGAATCGGGAACAC |  |  |  |
| *tet*(J) | tet(J)-F | ACAGACTCGCCAATCATTACGGTA | 300 | 55 | 85 |
|  | tet(J)-R | GCACCACCCAAAAAACCGAAAT |  |  |  |
| *tet*(M) | tet(M)-F | GTGGACAAAGGTACAACGAG | 406 | 55 | 84 |
|  | tet(M)-R | CGGTAAAGTTCGTCACACAC |  |  |  |
| *tet*(S) | tet(S)-F | CATAGACAAGCCGTTGACC | 667 | 55 | 84 |
|  | tet(S)-R | ATGTTTTTGGAACGCCAGAG |  |  |  |
| *tet*(X) | tet(X)-F | CAATAATTGGTGGTGGACCC | 468 | 55 | 84 |
|  | tet(X)-R | TTCTTACCTTGGACATCCCG |  |  |  |
| *tetP*(A) | tetP(A)-F | CTTGGATTGCGGAAGAAGAG | 676 | 55 | 84 |
|  | tetP(A)-R | ATATGCCCATTTAACCACGC |  |  |  |
| *tet*(X1) | tet(X1)-F | CGAAAAATGTTGCTTGGCAGCTT | 486 | 59 | 86 |
|  | tet(X1)-R | AGTTGTTGAACGAATTAACTCC |  |  |  |
| *tet*(X2) | tet(X2)-F | CGGGATGTCCAAGGTAAGAAAA | 343 | 59 | 86 |
|  | tet(X2)-R | TGACAACGTCGTATGAATCAA |  |  |  |
| *tet*(X3) | tet(X3)-F | GACACTTGATCTGCACAGGGATT | 685 | 59 | 86 |
|  | tet(X3)-R | CCCTACAAAAGATGATGTCAAAC |  |  |  |
| *tet*(X4) | tet(X4)-F | CTGATTCGTGTGACATCATCTTTTG | 204 | 59 | 86 |
|  | tet(X4)-R | GTTAAATTTCCCATTGGTCAGATTA |  |  |  |
| *tet*(X5) | tet(X5)-F | GGTATCAACATTTCAATGCTTG | 265 | 59 | 86 |
|  | tet(X5)-R | CGATTCGTCCTGCGTATCTTTTG |  |  |  |
| *sul1* | sul1-F | TCACCGAGGACTCCTTCTTC | 331 | 55 | 76 |
|  | sul1-R | CAGTCCGCCTCAGCAATATC |  |  |  |
| *sul2* | sul2-F | CCTGTTTCGTCCGACACAGA | 435 | 55 | 76 |
|  | sul2-R | GAAGCGCAGCCGCAATTCAT |  |  |  |
| *sul3* | sul3-F | GAGCAAGATTTTTGGAATCG | 790 | 51 | 40 |
|  | sul3-R | CATCTGCAGCTAACCTAGGGCTTTGGA |  |  |  |
